# Genotype and environment synergistically determine the polyphenolic composition and functional activity of highbush blueberries

**DOI:** 10.1038/s41598-025-23613-8

**Published:** 2025-11-13

**Authors:** Ireneusz Ochmian, Sabina Lachowicz-Wiśniewska, Miłosz Smolik

**Affiliations:** 1 Department of Horticulture, West Pomeranian University of Technology in Szczecin, Słowackiego 17 Street, 71-434 Szczecin, Poland; 2 Department of Medical and Health Sciences, University of Kalisz, W. Boguslawskiego 2 squer, 62-800 Kalisz, Poland; 3 Department of Biotechnology and Food Analysis, Wroclaw University of Economics and Business, Komandorska 118/120, 53-345 Wroclaw, Poland; 4 Department of Plant Genetics, Breeding and Biotechnology, West Pomeranian University of Technology in Szczecin, Słowackiego 17 Street, 71-434 Szczecin, Poland

**Keywords:** Nutraceutical potential, Bioactive compounds, Phytochemical profiling, Plant metabolomics, Cultivar and Somaclonal diversity, Plant sciences, Biogeochemistry, Health care

## Abstract

This study evaluated how genotype and origin shape the polyphenolic profile and antioxidant capacity of highbush blueberries grown under harmonized orchard conditions (19 genotypes, including somaclonal variants). Comprehensive profiling covered phenolic subclasses (anthocyanins, flavonols, phenolic acids, flavan-3-ols), antioxidant capacity (ABTS, DPPH, FRAP), and color (CIE L*), supported by multivariate analyses. Key results with numerical context. Among cultivars, *Brigitta* and *Draper* showed the highest totals of polyphenols (≈511.8–517.3) and anthocyanins (≈310.9–311.5). When comparing matched cultivar names by origin, German-sourced lines generally contained more anthocyanins and polyphenols than Polish-produced counterparts: for *Duke*, anthocyanins 208.4 vs 78.7–152.2 and polyphenols 401.2 vs 236.9–363.5; for *Patriot*, 286.9 vs 65.2–245.2 and 495.8 vs 293.5–409.0; for *Sunrise*, 258.8 vs 172.8–221.5 and 424.6 vs 342.0–411.1 (ANOVA/Tukey, p < 0.05 where indicated in tables). Antioxidant assays reflected these patterns with cultivar-dependent nuances: for example, *Duke* from Poland (DK10) showed higher ABTS (28.0) than the German source (11.1), whereas DPPH differences were modest for *Patriot* (≈13.8–14.1). Color supported the chemistry: Polish-produced fruits were lighter on average (higher CIE L*), consistent with lower anthocyanins. Genotype × origin interactions are decisive for blueberry chemotype and antioxidant properties. The quantitative differences reported here strengthen the case for authentication and source-aware selection in breeding, nursery supply, and raw-material procurement for functional food applications.

**Supplementary Information:**

The online version contains supplementary material available at 10.1038/s41598-025-23613-8.

## Introduction

Global production of highbush blueberry (*Vaccinium corymbosum* L.) amounts to nearly 655,000 tons annually and has increased 20-fold over the past two decades. The United States, China, Peru, Chile, and Canada are currently the leading producers, while in Europe, Poland, Germany, and Spain dominate production^1^. One of the major limiting factors in establishing new highbush blueberry plantations is the low availability of soils suitable for cultivation^2^.

In recent years, there has been growing interest in foods rich in bioactive compounds. Blueberries are considered a functional food due to their high polyphenolic content and strong antioxidant activity^3^. They are an excellent source of flavonoid compounds, including proanthocyanidins and flavonols, as well as non-flavonoid compounds such as hydroxycinnamic acid esters, with chlorogenic acid being especially abundant^4^.

Blueberries are available year-round as they are cultivated in many regions worldwide^5^, which allows consumers continuous access to their bioactive and prebiotic compounds, including polyphenols^6^. Among these, anthocyanins, flavonols, and phenolic acids are particularly important. Ochmian et al.^7^ showed that delphinidin and malvidin are the dominant anthocyanins in highbush and half-highbush blueberries, together accounting for approximately 75% of total anthocyanin content. Quercetin, on the other hand, is the main nutritionally significant flavonol, demonstrating up to 80% bioavailability during digestion.

Environmental factors strongly influence the chemical composition, sensory attributes, and phenotypic traits of blueberry fruits. Numerous studies confirm that soil type, pH, irrigation regime, light availability, and seasonal temperatures affect yield, firmness, sugar–acid balance, and polyphenol content, especially anthocyanins, flavonols, and phenolic acids. For example, Wang et al.^8^ demonstrated that greater day–night temperature variation (DTV) enhanced anthocyanin accumulation, while Kalt et al.^9^ and Connor et al.^10^ reported that fertilization and water status significantly shifted phenolic profiles.

Recent findings highlight the central role of genotype × environment × year (G × E × Y) interactions in shaping fruit quality. Gilbert et al.^11^ showed that seven biochemical parameters, including fructose and titratable acidity (TA), were significantly influenced by G × E × Y. Glucose exhibited substantial variation across genotypes (19%) and seasons (16%), while fructose was even more strongly affected by genetic factors (~ 30%). Fruit pH was largely determined by location (30%) and harvest year (6%). Volatile compounds such as 1-hexanol and E-2-hexenal showed the highest interannual variation, confirming the strong environmental modulation of blueberry aroma compounds.

Sater et al.^12^ also documented considerable variability in both sensory and instrumental traits—including firmness, sourness, and soluble solids content (SSC)—across six southern highbush blueberry cultivars over two growing seasons. They found that a ~ 1 °C increase in DTV prior to harvest raised SSC by up to 2.8°Brix, which reduced perceived sourness and green flavour. Conversely, heavy rainfall shortly before harvest decreased TA and firmness, although the magnitude of this effect varied among cultivars. Four-way interactions (cultivar × harvest × year × environment) were significant for most sensory and physicochemical traits, reflecting the complexity of environmental influences.

Lobos et al.^13^ studied the impact of delayed harvest on three late-season cultivars—’Aurora’, ‘Elliott’, and ‘Liberty’. Although TA consistently declined during ripening, SSC remained stable, resulting in sweeter fruit at later harvest dates. However, firmness and storage life decreased as fruit remained longer on the plant. Interestingly, ‘Aurora’ and ‘Liberty’ maintained better storability compared to ‘Elliott’, suggesting greater tolerance to delayed harvest without compromising postharvest quality. The strong correlation between instrumental and sensory data confirmed that SSC, TA, and firmness are reliable predictors of consumer acceptance.

These results align with shifting priorities in blueberry breeding programs, which now emphasise consumer-driven quality traits such as texture, size, appearance, and flavour, alongside agronomic traits like yield and ripening time^14,15^.

The rapid expansion of blueberry cultivation requires efficient propagation methods to meet the demand for seedlings. Traditionally, propagation has been achieved through hardwood, semi-hardwood, leafy, and root cuttings. However, this approach faces challenges such as low rooting percentages in many genotypes, long propagation cycles, and phytosanitary risks. Tissue culture offers an efficient alternative, allowing the production of virus-free and genetically uniform plants^16^. Successful *in vitro* propagation requires the selection of an appropriate medium and culture conditions^17,18^.

The quality of nursery material at the establishment stage has a lasting impact on fruit traits relevant to storage, processing, and distribution18. Nursery cultivar mix-ups—when plants are incorrectly labelled or placed—can lead to errors in cultivar identification, reducing fruit quality and undermining breeding efforts. In the case of patented cultivars, such errors may also cause legal and certification issues^19^.

When using *in vitro* techniques, it is important to maintain the genetic identity and stability of plant cultivars propagated by *in vitro *methods^19^. Somaclonal variability is often observed in long-term i*n vitro* cultures of berry plants. It is widely believed that somaclonal variation results from either permanent genetic changes or transient factors causing such changes. In *in vitro* cultures, the frequency of their occurrence is much higher, and under certain conditions, it can reach several percent per locus. The frequency of mutations depends on various factors: genotype, ploidy level, type of explants, regeneration system, medium composition, and culture duration^19^.

Under *in vitro* conditions, epigenetic variability is most observed, with changes affecting morphological traits (e.g., shape and surface structure of leaves, presence of thorns, or stem habit) or functional traits (e.g., increased shoot formation, delayed generative organ development, reduced fruit size, or changes in chemical composition). Since most changes at the DNA level are not phenotypically expressed^20,21^, it is necessary to use molecular methods to determine the actual degree of genetic variability in plants obtained via micropropagation compared to the parent plant^20^.

Somaclonal variation is inherently unpredictable, which poses challenges in maintaining trait stability during micropropagation. On the one hand, this is a limitation when the goal is to obtain standardized plant traits; on the other hand, it offers opportunities when the aim is to generate novel variability for breeding or genetic research.

Aware of the unique genotypes of *Vaccinium* sp., including somaclonal variants used in commercial blueberry production, we designed this study to describe the phenotypic variability observed within selected cultivars and their somaclonal derivatives. The experiment focused on three core cultivars—’Duke’ (DK, DK1, DK2, DK9, DK10), ‘Patriot’ (PT, PT1, PT2, PT9, PT10), and ‘Sunrise’ (SN, SN1, SN2, SN9, SN10)—to assess whether fruit quality remains consistent among plants of the same cultivar obtained from different nurseries. In addition, ‘Aurora’, [AUR] ‘Brigitta Blue’, [BRI] ‘Draper’ [DRA], and ‘Earliblue’ [EAR] were included as a reference group, providing a benchmark for fruit quality comparison.

The overarching aim of this study was to comprehensively assess how genotype, origin, and somaclonal variation influence the physical, chemical, and health-promoting properties of highbush blueberry (*Vaccinium corymbosum* L.) fruits. For this purpose, we analysed thirty-seven phenolic compounds (phenolic acids, flavonols, flavan-3-ols, and anthocyanins) alongside key physical traits (fruit colour, firmness, skin puncture resistance, soluble solids, and titratable acidity) and health-related parameters, including -*L*-ascorbic acid, nitrate and nitrite content, and antioxidant capacity (ABTS, DPPH, FRAP). This integrative approach enabled us to verify the stability of fruit quality within cultivars propagated in different nurseries and to identify differences between somaclonal variants and their parental genotypes, thereby providing new insights into the interaction between genotype × environment effects and somaclonal variation in shaping blueberry fruit quality.

The second objective, focusing on the genotypic characterization of selected somaclonal variants using molecular techniques, was addressed and published separately in another study.

## Results and discussion

For the nineteen highbush blueberry genotypes examined in this study, substantial variability was observed across several fruit traits. The mean, minimum, and maximum values, along with the standard deviation and coefficient of variation for each trait, are presented in the following tables. Relationships among these traits were analysed following the approach proposed by Grosser et al.^15^, who emphasised that the most effective test for assessing somaclonal variation is the evaluation of fruiting performance in somaclones. In the present study, fruits collected from fruiting shrubs of somaclones derived from different parental cultivars underwent a comprehensive horticultural assessment, which included detailed evaluations of selected physical and chemical fruit parameters. As noted by Gallardo et al.^22^, such an evaluation process is lengthy; however, maintaining fruiting somaclones of specific cultivars with known fruit characteristics within the plantation provides a considerable advantage. The unique gene pool analysed here thus represents valuable material for the development of new highbush blueberry cultivars.

Although variability among genotypes and somaclonal variants in traits such as colour intensity, firmness, soluble solids, or polyphenol concentration might initially appear to reflect cultivation environment effects, several factors suggest that genetic background and somaclonal variation played a dominant role. All shrubs were grown in a uniform peat-based substrate sourced from a single extraction site and transported to the experimental orchard. The substrate exhibited consistent physicochemical properties, including pH, organic matter content, and water retention capacity. Moreover, the experimental sites Ostoja and Reptowo are geographically close and experience nearly identical climatic conditions, including precipitation, temperature, and solar radiation.

Given the controlled substrate and comparable climatic environment, the observed trait differences are most reasonably attributed to genotype and *in vitro*-derived somaclonal effects rather than to macro-environmental variability. This interpretation is consistent with previous field-based studies, which demonstrate that although fruit quality parameters such as soluble solids content (SSC), titratable acidity (TA), firmness, and volatile profiles are modulated by environmental conditions, cultivar-specific responses remain relatively robust across seasons and locations^11,12^. Furthermore, even within late-season cultivars grown under similar conditions, ripening stage has been shown to exert a stronger influence on sensory and physicochemical traits than location per se^13^. In our study, the use of a standardised substrate and synchronised harvest timing minimised environmental variability, thereby strengthening the reliability of the observed genotypic differences, particularly in the context of somaclonal variation^13^.

The practical importance of these findings is underscored by evidence that even subtle differences in sugar–acid balance, firmness, and flavour perception can significantly shape consumer acceptance^10,15^. Accordingly, the differentiation observed between somaclones and their parental lines in traits such as soluble solids, acidity, and skin durability carries both biological relevance and commercial significance.

This approach aligns with contemporary breeding strategies, which increasingly emphasise validation of traits under semi-controlled yet field-representative conditions, rather than relying solely on greenhouse-based screening^14^. The results presented here are therefore consistent with the prevailing paradigm of identifying stable genotypic improvements through reproducible, field-level trials.

### Physical properties

Fruit colour. Significant differences in fruit colour were observed across genotypes depending on their origin (Table 1). Among the seven cultivars, the darkest fruits were found in DK and DK2, whereas the lightest were recorded in EAR, SN10, and PT10. These differences in fruit colour are strongly associated with variation in polyphenol content, particularly anthocyanins (Table 2). For example, DK and BRI exhibited both the darkest fruits and the highest anthocyanin levels, while EAR and DRA, which had lighter fruits, also showed significantly lower anthocyanin concentrations. Anthocyanins, as the principal pigments determining fruit colour, therefore play a central role in defining visual intensity. Their biosynthesis is further modulated by field conditions such as light exposure, temperature, and water availability^23^.

**Table 1:** Physical properties of blueberry (V*accinium corymbosum* L.) fruits.

Genotype	CIE L*	Firmness (G mm^–1^)	Puncture (G mm^–1^)	Total soluble solid (%Brix)	Titratable acidity (% in malic acid)
DK	28.93 ± 0.35^k^*	254.45 ± 11.22^gi^	91.90 ± 7.15^fi^	14.67 ± 0.42^fg^	0.61 ± 0.02^df^
DK1	32.70 ± 0.75^hj^	230.19 ± 13.81^i^	92.62 ± 5.10e^i^	14.50 ± 0.17^fh^	0.59 ± 0.01^eg^
DK2	28.23 ± 0.31^k^	250.64 ± 11.58^hi^	91,00 ± 12.72^gi^	16.23 ± 0.29^b^	0.47 ± 0.02^h^
DK9	33.17 ± 0.21^gj^	250.64 ± 5.50^hi^	81.67 ± 1.93^i^	15.40 ± 0.10^d^	0.54 ± 0.02^fh^
DK10	36.13 ± 0.67^df^	261.39 ± 2.16^ei^	87.27 ± 1.41^i^	16.80 ± 0.10^a^	0.72 ± 0.03^c^
PT	34.07 ± 0.51^fi^	284.96 ± 13.28^dg^	139.00 ± 13.00^ac^	13.17 ± 0.12^j^	0.70 ± 0.02^c^
PT1	35.67 ± 1.05^df^	261.39 ± 11.22^ei^	108.94 ± 12.15^dh^	12.77 ± 0.15^j^	0.73 ± 0.01^c^
PT2	36.17 ± 0.98^df^	228.80 ± 23.99^i^	93.71 ± 12.72^ei^	13.10 ± 0.10^j^	0.62 ± 0.01^de^
PT9	37.67 ± 0.67^bd^	307.49 ± 8.34^d^	146.25 ± 11.70^ab^	15.77 ± 0.15^bd^	0.54 ± 0.01^gh^
PT10	39.57 ± 0.93^ab^	257.23 ± 8.34^fi^	106.57 ± 9.93^di^	14.03 ± 0.12^hi^	0.61 ± 0.01^df^
SN	35.07 ± 0.67^eg^	394.85 ± 2.62^bc^	107.49 ± 2.57^dh^	16.03 ± 0.21^bc^	0.54 ± 0.02^gh^
SN1	34.67 ± 0.42^fh^	407.87 ± 7.27^b^	116.70 ± 4.48^cf^	15.33 ± 0.06^de^	0.59 ± 0.01^eg^
SN2	32.33 ± 0.42^ij^	288.43 ± 2.62^df^	107.60 ± 3.67^dh^	16.10 ± 0.10^b^	0.83 ± 0.04^b^
SN9	37.30 ± 0.30^cd^	271.23 ± 4.88^eh^	89.60 ± 1.60^gi^	14.87 ± 0.12^ef^	0.49 ± 0.02^h^
SN10	38.63 ± 0.32^ac^	415.81 ± 10.86^ab^	117.40 ± 4.78^ce^	15.50 ± 0.10^d^	0.61 ± 0.03^dg^
AUR	34.13 ± 0.57^fi^	369.32 ± 12.79^c^	134.90 ± 3.38^ac^	17.07 ± 0.12^a^	1.04 ± 0.05^a^
BRI	31.77 ± 1.11^j^	446.17 ± 15.74^a^	150.97 ± 7.77^a^	15.57 ± 0.21^cd^	0.67 ± 0.04^cd^
DRA	36.90 ± 0.17^ce^	294.32 ± 7.28^de^	114.26 ± 12.71^cg^	13.70 ± 0.10^i^	0.57 ± 0.02^eg^
EAR	39.93 ± 1.40^a^	237.81 ± 7.08^i^	123.38 ± 4.58^bd^	14.20 ± 0.10^gi^	0.54 ± 0.02^gh^
Mean ± SD	34.90 ± 3.22	300.68 ± 68.87	110.59 ± 21.48	14.99 ± 1.24	0.63 ± 0.13
Min–Max	27.90–43.06	198.00–462.80	71.00–163.80	12.60–17.20	0.46–1.09
CV%	9	23	19	8	21

*significant differences in the same column are represented by different letters (p < 0.05); values are means ± standard deviation.

**Table 2:** Polyphenol compound concentrations in different *Vaccinium corymbosum* L. genotypes expressed in g/100 g fresh matter (fm).

Genotype	Phenolic acid	Flavonols	Flavan-3-ols	Anthocyanins	Polyphenols
DK	142.03 ± 0.47^ h^*	22.51 ± 0.48^e^	28.29 ± 0.03^ l^	208.37 ± 0.22^ h^	401.21 ± 0.20^ h^
DK1	145.19 ± 1.06^ g^	20.55 ± 0.15^hi^	35.85 ± 0.04^e^	207.73 ± 0.33^ h^	409.32 ± 1.20^ g^
DK2	163.84 ± 1.43^b^	32.83 ± 0.03^a^	57.56 ± 0.56^a^	202.35 ± 0.57^i^	456.57 ± 0.33^e^
DK9	152.91 ± 1.14^ed^	18.33 ± 0.08^ k^	40.13 ± 0.09^d^	152.15 ± 0.07^ l^	363.52 ± 1.22^j^
DK10	110.97 ± 0.59^ k^	21.33 ± 0.01f.	25.90 ± 0.03^ m^	78.72 ± 0.05^n^	236.91 ± 0.51^n^
PT	154.50 ± 1.51^ cd^	28.01 ± 0.22^b^	26.34 ± 0.06^ m^	286.92 ± 1.08^b^	495.77 ± 0.15^c^
PT1	144.42 ± 1.09^hg^	20.32 ± 0.03^i^	30.64 ± 0.24^i^	176.93 ± 1.29^j^	372.31 ± 0.01^i^
PT2	104.79 ± 3.53^ l^	20.77 ± 0.08^gh^	23.46 ± 0.04^n^	214.94 ± 0.01^ g^	371.30 ± 3.38^i^
PT9	128.00 ± 0.40^i^	19.17 ± 0.01^j^	16.24 ± 0.05^o^	245.22 ± 0.66^d^	408.95 ± 1.12^ g^
PT10	169.11 ± 0.93^a^	19.07 ± 0.07^j^	40.10 ± 0.13^d^	65.17 ± 0.01^o^	293.45 ± 0.73^ m^
SN	99.15 ± 0.20^ m^	26.14 ± 0.08^c^	40.49 ± 0.07^d^	258.83 ± 0.20^c^	424.60 ± 0.14f.
SN1	110.49 ± 0.51^ k^	21.15 ± 0.06^ fg^	41.48 ± 0.20^c^	230.36 ± 0.06^e^	403.48 ± 0.72^ h^
SN2	161.61 ± 0.50^b^	22.32 ± 0.22^e^	32.58 ± 0.22^ g^	248.06 ± 4.67^d^	464.57 ± 3.73^d^
SN9	131.14 ± 0.68^i^	23.45 ± 0.07^d^	35.06 ± 0.19f.	221.46 ± 0.25f.	411.11 ± 0.69^ g^
SN10	121.83 ± 1.02^j^	18.50 ± 0.04^ k^	28.87 ± 0.04^ k^	172.81 ± 0.25^ k^	342.00 ± 0.77^ l^
AUR	148.63 ± 0.67f.	21.49 ± 0.08f.	43.85 ± 0.22^b^	143.68 ± 0.38^ m^	357.66 ± 0.01^ k^
BRI	144.75 ± 1.20^hg^	25.86 ± 0.28^c^	29.73 ± 0.02^j^	311.46 ± 1.60^a^	511.79 ± 0.15^b^
DRA	151.33 ± 0.44^fe^	23.24 ± 0.12^d^	31.79 ± 0.07^ h^	310.90 ± 1.30^a^	517.25 ± 1.93^a^
EAR	157.12 ± 0.97^c^	20.46 ± 0.02^hi^	23.01 ± 0.03^n^	200.85 ± 0.92^i^	401.44 ± 1.88^ h^
Mean ± SD	139.44 ± 20.06	22.39 ± 3.57	33.23 ± 9.20	207.21 ± 65.52	402.27 ± 69.15
Min–Max	98.88–170.34	18.23–32.87	16.17–58.13	65.16–313.10	236.39–519.23
CV%	14.39	15.96	27.67	31.62	17.19

*different letters in the same column indicate statistically significant differences (P ≤ 0.05); values are means ± standard deviation.

Fruits of DK, PT, and SN cultivars harvested from Germany were darker compared to their counterparts cultivated in nurseries in Uroczysko and Ciepłucha, where the lightest fruits were consistently observed (Table 1). Within somaclones, additional differences emerged: the DK1 somaclone produced lighter fruits than DK and DK2, while SN2 fruits were darker than SN and SN1. These variations may possibly result from somaclonal mutations affecting the content and composition of polyphenolic compounds^24^. Micropropagation processes are known to induce subtle shifts in plant metabolism, which can alter anthocyanin and other phenolic levels, thereby influencing fruit colour^25^. Thus, variation in blueberry fruit colour reflects both genetic and environmental contributions, as supported by prior studies demonstrating the influence of plant material origin on colour intensity^26,27^.

Fruit firmness and skin puncture resistance. Firmness and skin puncture resistance are closely related physical traits with significant implications for fruit quality and postharvest performance. Firmness reflects the structural integrity of the fruit flesh, whereas puncture resistance indicates the mechanical resilience of the skin. BRI fruits demonstrated the highest firmness values and correspondingly strong puncture resistance (Table 1). This combination suggests denser tissues in both skin and flesh, which enhances durability and reduces susceptibility to mechanical damage during storage and transport—traits especially valuable for the fresh fruit market^27^. In contrast, DK fruits, which are inherently characterised by low firmness, also exhibited low puncture resistance. These results are consistent with earlier findings by Saftner et al.^28^, who reported that softer fruits are generally more prone to skin injury during handling. Environmental factors such as high sunlight exposure and adequate irrigation are known to enhance skin thickness and toughness, thereby improving these parameters^29,30^. Interestingly, DK and SN fruits from Uroczysko displayed improved firmness and puncture resistance compared with those from other nurseries, whereas somaclones generally showed reduced firmness relative to their parental cultivars (Table 1). According to Cellon et al.^31^, genotypes expressing such favourable agronomic traits can be effectively utilised in breeding programmes.

Total soluble solids (TSS) content and acidity. TSS content and titratable acidity are key determinants of flavour balance in blueberries (Table 1). TSS includes sugars, organic acids, minerals, and soluble compounds contributing to fruit sweetness, while acidity defines sharpness and freshness. AUR fruits exhibited both the highest extract (17.07%) and highest acidity (1.04%), producing an intense flavour characterised by a combination of sweetness and tanginess. Such a sugar–acid balance is often preferred by consumers seeking more pronounced taste complexity^32^. In contrast, PT fruits had the lowest TSS (13.17%) and relatively low acidity (0.70%), resulting in a milder and less distinctive flavour profile, which may appeal to consumers preferring softer taste attributes^28^. These differences can be attributed not only to genotype but also to environmental influences such as light, temperature, and irrigation, which strongly regulate sugar and organic acid biosynthesis^33^. DK and PT fruits originating from the Uroczysko and Ciepłucha nurseries exhibited significantly higher TSS levels than their counterparts from other sites. Moreover, somaclones demonstrated both favourable and unfavourable shifts in sugar and acid content compared with their parental cultivars, consistent with the potential impact of somaclonal mutations on metabolic traits^34^.

### Health-promoting properties

The studied highbush blueberry cultivars exhibited substantial variability in the content of harmful compounds (nitrates and nitrites), health-promoting compounds (*L*-ascorbic acid), and antioxidant activity (Table 3). Among the cultivars, DRA and BRI appear particularly beneficial for human health due to their high *L*-ascorbic acid content combined with low nitrate and nitrite levels. The presence of nitrites (NO₂⁻) and nitrates (NO₃⁻) in fruits is strongly influenced by growing conditions, particularly nitrogen fertilization^35^. Nitrates occur naturally in many fruits and vegetables as a consequence of plant physiological processes. Although nitrates and nitrites are common dietary components, the levels recorded in blueberries are relatively low compared with other fruits and especially with vegetables typically associated with high nitrate accumulation^35–37^. According to current EU regulations, nitrate content is regulated only for leafy green vegetables (up to 5000 mg/1000 g in fresh lettuce), while processed foods for infants and young children must not exceed 200 mg/1000 g. Nitrite intake should remain below 0.07 mg per kg of body weight daily (EC, 2005). The values observed in blueberries in this study were far below these thresholds, confirming their safety for consumers.

**Table 3:** Health-promoting properties of blueberry (*Vaccinium corymbosum* L.) fruits.

Genotype	*L-*ascorbic acid (% fm)	NO_2_^-^ (mg/1000 g fm)	NO_3_^-^ (mg/1000 g fm)	ABTS (mmTe/100 g fm)	DPPH (mmTe/100 g fm)	FRAP (mmTe/100 g fm)
DK	83.00 ± 5.29^eg^*	0.22 ± 0.01^a^	37.43 ± 0.25^ l^	11.10 ± 0.10^ij^	17.87 ± 0.15^d^	20.40 ± 0.26^a^
DK1	82.33 ± 13.0^eg^	0.19 ± 0.01^bd^	44.23 ± 0.25^j^	10.37 ± 0.12^jl^	18.13 ± 0.15^d^	17.33 ± 0.38^b^
DK2	95.00 ± 3.61^de^	0.17 ± 0.02^df^	45.77 ± 0.49^i^	8.27 ± 0.06^ m^	15.60 ± 0.10f.	14.47 ± 0.31^d^
DK9	78.67 ± 7.77^fh^	0.21 ± 0.01^ac^	31.77 ± 0.31^n^	15.67 ± 0.12^e^	18.27 ± 0.38^d^	17.67 ± 0.15^b^
DK10	54.00 ± 4.58^ij^	0.24 ± 0.02^a^	73.17 ± 0.40^b^	27.97 ± 1.14^a^	20.37 ± 0.23^ac^	17.80 ± 0.17^b^
PT	65.67 ± 3.2^hi^	0.22 ± 0.01^ab^	42.90 ± 0.36^ k^	10.60 ± 0.10^ik^	13.83 ± 0.38^ g^	11.30 ± 0.17f.
PT1	47.67 ± 0.58^j^	0.21 ± 0.01^ab^	38.37 ± 0.40^ l^	15.83 ± 0.38^e^	20.17 ± 0.21^bc^	15.27 ± 0.21^ cd^
PT2	69.67 ± 3.5^gh^	0.16 ± 0.01^df^	45.33 ± 0.21^ij^	11.53 ± 0.12^hi^	17.13 ± 0.15^e^	14.37 ± 0.06^d^
PT9	84.00 ± 40.0^dg^	0.15 ± 0.01^ef^	49.50 ± 0.17^ g^	12.40 ± 0.30^gh^	14.07 ± 0.21^ g^	9.40 ± 0.26^ h^
PT10	42.33 ± 1.53^j^	0.24 ± 0.02^a^	69.57 ± 0.60^c^	9.67 ± 0.21^kl^	11.40 ± 0.17^i^	15.93 ± 0.47^c^
SN	77.33 ± 4.93^fh^	0.09 ± 0.01^g^	35.53 ± 0.32^ m^	17.47 ± 0.21^ cd^	20.43 ± 0.21^ab^	11.27 ± 0.21f.
SN1	83.00 ± 20.0^eg^	0.10 ± 0.01^g^	42.90 ± 0.36^ k^	16.43 ± 0.32^de^	21.07 ± 0.35^a^	10.80 ± 0.30^ fg^
SN2	114.67 ± 5.51^ab^	0.22 ± 0.01^a^	84.00 ± 0.46^a^	11.13 ± 0.32^ij^	12.50 ± 0.17^ h^	9.90 ± 0.30^gh^
SN9	91.67 ± 1.53^cf^	0.14 ± 0.01^ef^	54.17 ± 0.15f.	18.33 ± 0.12^c^	17.77 ± 0.21^de^	12.47 ± 0.74^e^
SN10	104.67 ± 2.52^bc^	0.16 ± 0.02^df^	49.43 ± 0.12^ g^	14.43 ± 0.06f.	15.27 ± 0.06f.	13.07 ± 0.21^e^
AUR	71.33 ± 1.53^gh^	0.18 ± 0.01^cd^	66.50 ± 0.53^d^	22.53 ± 0.55^b^	19.67 ± 0.21^c^	14.77 ± 0.46^d^
BRI	112.33 ± 3.51^ab^	0.14 ± 0.01f.	47.23 ± 0.25^ h^	18.40 ± 0.26^c^	18.03 ± 0.12^d^	8.10 ± 0.10^i^
DRA	125.67 ± 7.57^a^	0.10 ± 0.01^g^	35.30 ± 0.78^ m^	13.20 ± 0.20^ g^	15.67 ± 0.38f.	10.40 ± 0.17^ fg^
EAR	99.33 ± 4.04^bcd^	0.17 ± 0.01^de^	59.30 ± 0.56^e^	9.47 ± 0.21^ l^	15.20 ± 0.10f.	17.47 ± 0.38^b^
Mean ± SD	83.28 ± 22.41	0.17 ± 0.05	50.13 ± 14.05	14.46 ± 4.91	16.97 ± 2.77	13.80 ± 3.38
Min–Max	41.00–131.00	0.08–0.25	31.50–84.50	8.20–28.90	11.20–21.40	8.00–20.60
CV%	27	26	28	34	16	24

*different letters in the same column indicate statistically significant differences (P ≤ 0.05); values are means ± standard deviation.

Interestingly, plant origin significantly influenced nitrate and nitrite concentrations. Fruits from DK and PT genotypes sourced from Germany contained moderate levels, while SN fruits showed the lowest nitrite levels of all genotypes. In contrast, DK10 and PT10 fruits (Uroczysko nursery) accumulated higher concentrations, whereas genotypes grown in the Ciepłucha nursery (DK9, SN9) showed consistently lower levels. Somaclones frequently exhibited higher nitrite and nitrate levels than their parental genotypes, with SN2 standing out for its elevated values of both compounds. These differences likely reflect not only fertilization practices and soil nitrogen cycling^19^ but also the effect of cultivation on peat-based substrates. Peat soils, rich in organic matter, promote nitrate accumulation, whereas sandy soils are prone to nitrate leaching, thereby reducing concentrations^28^.

Antioxidant activity. Antioxidant properties assessed using ABTS, DPPH, and FRAP assays, also varied significantly among cultivars (Table 3). PT, EAR, and BRI demonstrated the highest antioxidant capacity, suggesting greater potential health benefits, while AUR and SN showed lower activity, particularly in ABTS and DPPH. Fruits from Uroczysko nursery frequently exhibited stronger antioxidant activity than those from Germany, underlining the influence of plant origin even under comparable growing conditions. Somaclonal variation further modulated these traits: DK2 and SN2 displayed markedly improved antioxidant properties relative to their parents, whereas PT1 showed reduced capacity. These findings emphasize the importance of evaluating individual somaclones when selecting planting material with desirable health-promoting traits.

A positive correlation was observed between total polyphenol content and antioxidant activity across the cultivars studied. Genotypes rich in polyphenols, such as BRI and DRA, consistently exhibited higher antioxidant activity, underscoring the central role of polyphenols in determining antioxidant potential^38^. Other bioactive compounds, notably *L*-ascorbic acid, also contributed to antioxidant properties, further enhancing the health-promoting value of these fruits^39^.

### Polyphenols compounds concentrations

Analyzing the content of 37 polyphenolic compounds classified into phenolic acids, flavonols, flavan-3-ols, and anthocyanins, significant differences in their concentration were demonstrated depending on the cultivars, their origin, the somaclonal variant, and the genotype from which it was derived (Table 2 and Table S1). The observed variation between cultivars may significantly influence the evaluation of their health-promoting properties, including their antioxidant capacities.

It was shown that DRA, BRI, and PT stood out with the highest total polyphenol content, mainly due to their high anthocyanin and flavonol levels (Table 2). In contrast, AUR exhibited the lowest total polyphenol content, primarily due to its low anthocyanin concentration (Table 2).

Attention was drawn to the fact that fruits harvested from the DK, PT, and SN cultivars originating from Germany had high polyphenol content, particularly anthocyanins. In the same cultivars (DK10, PT10, and SN10), but grown at the Uroczysko nursery in Poland, the lowest polyphenol content, especially anthocyanins, was observed. The low levels of these compounds in their counterparts (DK10, PT10, and SN10) may indicate a lower health-promoting potential than BRI or DRA. It is known that anthocyanins accumulate mainly in the fruit skin, giving it a blue colour. The light coloration of the fruit skin in the DK10, PT10, and SN10 cultivars is consistent with the relationship described by Ribera et al.^40^.

When analyzing the polyphenol content within somaclones, it was found that DK2 and SN2 had higher total polyphenol content than the cultivars from which they were derived. On the other hand, the somaclones PT1 and PT2, as phenotypic outcomes of the Patriot cultivar, had significantly lower polyphenol content. Detailed data on the content of phenolic acids, flavonols, flavan-3-ols, and anthocyanins, not only for the somaclones, are presented in Table 2, while individual polyphenol levels are listed in Table S1.

The scientific literature contains numerous reports on the existence of somaclonal variation during *in vitro* propagation. The causes of its emergence, phenotypic and genotypic effects, and reports on its influence on processes like the creation of new cultivars are well described in the work of Krishna et al.^41^.

Regarding polyphenols per se, it has been shown that various cultivation methods, irrigation, and sunlight exposure can influence their content in fruits^27^. De Klerk^42^ highlights the existence of differences in the genetic profiles of somaclones and the potential impact of these differences on the ability to synthesize bioactive compounds such as anthocyanins, phenolic acids, flavonols, and flavan-3-ols. Smulders and de Klerk^42^ argue that somaclones can exhibit both positive and negative secondary metabolite production profiles, depending on the type of spontaneous mutations occurring during the regeneration process.

We demonstrated that anthocyanins dominated the entire profile of polyphenolic compounds in highbush blueberry fruits (Table 2 and Table S1). The most striking variability was revealed in the anthocyanin profile, which determines fruit colour. For instance, the highest malvidin-3-galactoside content (around 90 mg/100 g FW) was found in BRI and DRA, while the lowest was recorded in PT10 (11.25 mg/100 g FW). High variation in the content of this specific compound has also been reported in other studies^43,44^, and similar variability was typical for other anthocyanins. Notably, PT accumulated the highest levels of delphinidin-3-*O*-glucoside (70.23 mg/100 g FW), while SN was distinguished by its elevated cyanidin-3-*O*-glucoside concentration (65.88 mg/100 g FW). These findings indicate that genotypes and their somaclones exhibit distinct anthocyanin “fingerprints,” although establishing linear relationships between plant origin and specific compound content proved difficult. As anthocyanins are key pigments in blueberries, their high variability has major implications not only for sensory attributes but also for health-promoting effects, including protection against cardiovascular disease, diabetes, and cancer^45^. Moreover, polyphenols, including anthocyanins, play a central role in plant defense mechanisms^46^.

Beyond anthocyanins, substantial variation was also observed in phenolic acids, where chlorogenic acid was the dominant metabolite. Its content ranged from 53.08 mg/100 g FW (PT2) to *157.97 mg/100 g FW (DK2), consistent with previous reports by Ochmian et al.^47^ Genotypes such as EAR and PT also showed high chlorogenic acid levels (149.10 and 144.57 mg/100 g FW, respectively), confirming strong genotypic differentiation in the accumulation of this quality marker. The SN group exhibited a unique profile, characterized by the highest neochlorogenic acid (4.46 mg/100 g FW) and cryptochlorogenic acid (4.05 mg/100 g FW), while SN10 accumulated minimal amounts (1.55 and 0.10 mg/100 g FW, respectively).

Within the flavonol group, particularly wide variation was recorded among quercetin derivatives. PT showed the highest accumulation of quercetin-3-galactoside (10.57 mg/100 g FW) and myricetin-3-galactoside (6.52 mg/100 g FW), whereas BRI contained the greatest concentration of quercetin-3-rhamno-hexoside (4.40 mg/100 g FW). SN had the maximum quercetin-3-rutinoside level (2.80 mg/100 g FW), while DK2 was notable for its exceptionally high quercetin-3-oxalylpentoside (8.45 mg/100 g FW). These results highlight that each genotype possesses a unique “chemical signature,” which may serve as a marker for cultivar identification and differentiation.

The flavan-3-ol group also showed remarkable variability. A surprising finding was the exceptionally high catechin content (37.47 mg/100 g FW) in the somaclonal variant DK2, while DK10 contained only 4.53 mg/100 g FW. Similarly, AUR accumulated elevated catechin levels (25.49 mg/100 g FW). In terms of (-)-epicatechin, DK1 (8.23 mg/100 g FW) and DRA (8.18 mg/100 g FW) were the leading genotypes. Additionally, PT10 displayed an exceptionally high concentration of a specific procyanidin dimer (17.47 mg/100 g FW), indicating selective accumulation of certain oligomers. These flavan-3-ols are known for their vascular protective properties, contributing to improved elasticity and reduced risk of atherosclerosis^48^.

Taken together, our results demonstrate that highbush blueberry genotypes and their somaclonal variants exhibit distinct polyphenolic fingerprints. BRI and DRA were dominated by malvidin derivatives, PT by delphinidin and quercetin compounds, SN by cyanidin and phenolic acids (neochlorogenic and cryptochlorogenic), and DK2 by chlorogenic acid, catechin, and unique quercetin glycosides. This diversity directly translates into the sensory properties of fruits (colour, taste, astringency), as well as their nutritional and health-promoting potential.

Studies on genotype × environment (G × E) interactions in blueberries have confirmed that the content of antioxidant polyphenols, such as anthocyanins and phenolic acids, differs significantly among cultivars depending on environmental conditions^49^. Factors such as sunlight, temperature, water availability, and cultivation practices play a key role in anthocyanin biosynthesis and thus influence fruit colour and antioxidant potential^47^. The genotypic diversity in response to these factors underlines the importance of G × E interactions in shaping the phenolic profile and commercial quality of fruits.

The propagation method is another important factor influencing plant and fruit traits. Conventional vegetative propagation ensures progeny uniformity but is time-consuming, weather-dependent, and limited by the availability of mother plants^50,51^. It therefore does not meet the increasing demand for highbush blueberry seedlings. Micropropagation provides an efficient alternative, allowing rapid plant multiplication under controlled laboratory conditions, independent of season and weather^52^. However, this technique is not free from limitations, with somaclonal variation being one of the main challenges. This phenomenon involves genetic and epigenetic changes arising during *in vitro* culture^53–55^, which may result from both pre-existing variability among explants and culture-induced factors such as medium composition, growth regulator type and concentration, passage duration, and regeneration pathway^56–58^. Adventitious shoots, common in highbush blueberry cultures, are particularly prone to somaclonal changes and difficult to distinguish from axillary shoots, which increases the risk of unintentional selection of mutated or epigenetically altered lines^59^.

The consequences of somaclonal variation can be both positive and negative. On the one hand, plants regenerated *in vitro* may show delayed fruiting, reduced yields, or smaller fruit size^60,61^ On the other hand, beneficial traits such as enhanced rooting, vigorous growth, and stronger branching have also been reported^62^. In highbush blueberry, Debnath and Ghosh^63^ demonstrated that plants derived from tissue culture exhibited higher antioxidant levels compared to conventionally propagated plants, suggesting a potential influence of somaclonal changes on polyphenol metabolism. Other studies have confirmed differences in total phenolic content and antioxidant activity^53^, supporting the view that somaclonal variation can directly affect secondary metabolism, including anthocyanin biosynthesis. Since anthocyanins and other polyphenols determine both fruit colour and health-promoting potential, such differences are of considerable practical importance.

The mechanisms underlying somaclonal variation include both genetic mutations and epigenetic modifications, particularly DNA methylation changes, which regulate gene expression and metabolic pathways. MSAP analysis has revealed substantial variability in DNA methylation among regenerants^64,65^, highlighting the significant role of epigenetic regulation in shaping somaclonal phenotypes and secondary metabolism^66^.

Integrating the effects of G × E interactions with somaclonal variation is essential for a comprehensive understanding of blueberry biology. While environmental factors strongly modulate polyphenol biosynthesis^49^, processes occurring during *in vitro* culture may further amplify or alter these effects. The result is a wide spectrum of biochemical variability, which, although challenging for maintaining uniform seedling quality, offers opportunities for selecting genotypes with desirable traits such as higher anthocyanin content or antioxidant activity. Somaclonal variation, if properly understood and managed, can thus be harnessed as a valuable tool in breeding programs aimed at developing cultivars with enhanced functional and health-promoting properties. Previous studies on *Vaccinium* have focused mainly on morphological and growth-related traits, while biochemical and epigenetic aspects remain less explored^60,67^, further emphasizing the novelty and importance of the present research.

### Correlations

Fruits with darker coloration (i.e., those with lower CIE L values) exhibit higher antioxidant activity (Tables S2 and S3). They are typically richer in polyphenols^7^, especially anthocyanins and flavonols. Firm fruits are more resistant to mechanical damage and often contain higher levels of *L*-ascorbic acid. Increased sugar content and higher acidity are associated with enhanced antioxidant potential, as observed with polyphenolic compounds, particularly anthocyanins. Therefore, polyphenols play a crucial role in the health-promoting properties of fruits^7^.

A detailed correlation analysis (Table S2) revealed strong associations between fruit colour, phenolic composition, and antioxidant capacity. A significant negative correlation was observed between CIE L values and several polyphenolic groups, including flavonols (r = –0.58), flavan-3-ols (r = –0.45), anthocyanins (r = –0.25), and total polyphenols (r = –0.39). These results confirm that fruits with darker skin (lower CIE L) accumulate higher levels of bioactive compounds, consistent with the well-known relationship between anthocyanin pigmentation and antioxidant potential in blueberries. The negative correlation between CIE L and DPPH activity (r = –0.22) further suggests that darker fruits also exhibit stronger free radical scavenging activity.

Notably, anthocyanins demonstrated a very strong positive correlation with total polyphenol content (r = 0.93) and a moderate correlation with flavonols (r = 0.45), highlighting their predominant role in shaping the blueberry polyphenolic profile. These relationships indicate that anthocyanins are not only major contributors to fruit colour but also strongly determine overall antioxidant potential. Flavonols were positively correlated with both flavan-3-ols (r = 0.45) and anthocyanins (r = 0.45), which may point to shared regulatory pathways in their biosynthesis.

The comparison of antioxidant assays further underlined mechanistic differences. ABTS and DPPH assays were strongly correlated (r = 0.67), confirming their consistency in reflecting radical scavenging capacity. In contrast, FRAP showed significant negative correlations with anthocyanins (r = –0.68) and total polyphenols (r = –0.61), suggesting that this assay captures reducing activity linked to other compounds, possibly phenolic acids or ascorbic acid, rather than anthocyanins. This discrepancy illustrates that the choice of assay influences the interpretation of antioxidant potential, as each method targets different reaction mechanisms.

Additional correlations between biochemical and physical traits provide further mechanistic insights. Firmness was positively associated with puncture resistance (r = 0.59), and puncture resistance itself correlated positively with anthocyanin (r = 0.44) and total polyphenol content (r = 0.40). This indicates that berries with stronger mechanical properties also tend to be richer in polyphenols, potentially enhancing both storability and nutritional value. *L*-ascorbic acid content was strongly correlated with anthocyanins (r = 0.66) and total polyphenols (r = 0.67), suggesting synergistic accumulation of vitamin C and phenolics in fruits with higher antioxidant potential.

Finally, nitrogen compounds were linked with both biochemical and mechanical traits. Nitrate (NO₃⁻) levels correlated positively with acidity (r = 0.51) but negatively with DPPH activity (r = –0.36), whereas nitrite (NO₂⁻) levels correlated negatively with firmness (r = –0.56), *L*-ascorbic acid (r = –0.50), anthocyanins (r = –0.60), and total polyphenols (r = –0.46). At the same time, NO₂⁻ correlated positively with FRAP (r = 0.59) and phenolic acids (r = 0.48). This pattern may indicate that nitrite accumulation is associated with metabolic shifts reducing anthocyanin biosynthesis and fruit firmness, while enhancing antioxidant activity via alternative compounds detectable by FRAP.

Taken together, these correlations provide strong evidence that fruit colour intensity, physical integrity, and biochemical composition are tightly interrelated. Darker, firmer berries not only contain higher concentrations of anthocyanins and other polyphenols but also show superior antioxidant activity in radical scavenging assays. However, divergent responses across ABTS, DPPH, and FRAP underline the complexity of antioxidant mechanisms, reflecting contributions from distinct compound classes.

The firmness of the fruits was positively correlated with puncture resistance (r = 0.59), indicating that firmer fruits are also more resistant to mechanical damage. This finding is consistent with the earlier observations of Ochmian et al.^37^ Moreover, puncture resistance was positively correlated with both anthocyanin content (r = 0.44) and total polyphenol content (r = 0.40), suggesting that fruits with stronger structural integrity tend to accumulate higher levels of these compounds, which in turn contribute to enhanced antioxidant potential.

Soluble solids content, expressed as extract percentage, was positively associated with antioxidant activity measured by ABTS (r = 0.51), indicating that fruits with higher sugar levels may also possess stronger radical-scavenging capacity. Acidity, on the other hand, showed a positive correlation with nitrate content (r = 0.51), suggesting that higher titratable acidity could be linked to increased nitrate accumulation in the fruits.

L-ascorbic acid content demonstrated strong positive correlations with anthocyanins (r = 0.66) and total polyphenols (r = 0.67), and a moderate correlation with flavonols (r = 0.22). These relationships highlight that fruits richer in vitamin C also tend to accumulate higher concentrations of polyphenolic compounds, thereby reinforcing their antioxidant potential.

Among the potentially harmful compounds, nitrate (NO₃⁻) levels were positively correlated only with titratable acidity (r = 0.51) and showed a moderate negative correlation with DPPH antioxidant activity (r = –0.36). In contrast, nitrite (NO₂⁻) content was negatively correlated with firmness (r = –0.56) and *L*-ascorbic acid (r = –0.50), while positively correlated with FRAP activity (r = 0.59) and phenolic acids (r = 0.48). Furthermore, nitrite content showed significant negative correlations with both total anthocyanins (r = –0.60) and total polyphenols (r = –0.46). These patterns suggest that higher nitrite accumulation may be associated with softer fruits, lower vitamin C, and reduced polyphenol levels, potentially reflecting stress-related shifts in cellular metabolism.

Antioxidant activity measured using ABTS, DPPH, and FRAP assays showed complex associations with the polyphenolic profile. ABTS and DPPH were moderately correlated with each other (r = 0.67), suggesting consistency between these two assays in capturing radical-scavenging activity. Anthocyanins, the dominant group of polyphenols in blueberries, exhibited a very strong positive correlation with total polyphenols (r = 0.93) and a moderate correlation with flavonols (r = 0.58), confirming their central role in shaping the antioxidant profile of the fruit. In contrast, FRAP activity was negatively correlated with anthocyanins (r = –0.68) and total polyphenols (r = –0.61), implying that FRAP may capture antioxidant contributions from alternative redox-active compounds rather than anthocyanins. Similarly, phenolic acids showed negative correlations with both ABTS (r = –0.51) and DPPH (r = –0.60), underlining that the contribution of individual polyphenol groups to antioxidant activity depends on the assay applied.

Further insights were provided by compound-specific correlations (Table S3). Fruits with lighter coloration (higher CIE L values) produced lower concentrations of anthocyanins, particularly cyanidin-3-*O*-glucoside and delphinidin-3-*O*-glucoside, with correlations of –0.23 and –0.15, respectively. Conversely, firmness was positively correlated with cyanidin-3-*O*-glucoside (r = 0.36) and delphinidin-3-*O*-glucoside (r = 0.27), suggesting that anthocyanin accumulation contributes to fruit structural integrity. Puncture resistance correlated positively with malvidin-3-*O*-glucoside (r = 0.50), further linking anthocyanin content with mechanical resilience.

Relationships between flavonols and fruit quality traits were also evident. Extract content correlated positively with quercetin-3-glucoside (r = 0.60) and quercetin-3-(6′-acetyl)galactoside (r = 0.50), indicating that fruits with higher sugar content are also richer in specific quercetin derivatives. Acidity was inversely correlated with caffeoyl glucose (r = –0.36) and chlorogenic acid (r = –0.29), suggesting that higher organic acid levels may reduce the accumulation of hydroxycinnamic acids.

Interestingly, FRAP values were negatively correlated with key anthocyanins such as malvidin-3-*O*-glucoside (r = –0.64) and petunidin-3-*O*-glucoside (r = –0.57), further confirming that FRAP reflects antioxidant mechanisms distinct from those measured by DPPH and ABTS. At the same time, DPPH and ABTS values showed positive correlations with quercetin aglycone and quercetin-3-rhamno-hexoside, suggesting that these compounds contribute strongly to radical scavenging activity in these assays.

Finally, several flavonoid compounds showed co-accumulation patterns, indicating shared metabolic pathways. For example, quercetin-3-arabinoside correlated positively with malvidin-3-galactoside (r = 0.51), while quercetin-3-galactoside showed very strong positive correlations with cyanidin-3-*O*-glucoside (r = 0.86) and petunidin-3-*O*-glucoside (r = 0.88). These relationships suggest coordinated biosynthetic regulation of quercetin derivatives and anthocyanins, likely mediated by common transcriptional or enzymatic controls.

Taken together, these results demonstrate that blueberry fruit quality results from a complex interplay of polyphenolic composition, mechanical properties, and antioxidant activity. Importantly, the observed correlations reveal that anthocyanins and flavonols are central determinants of both colour and antioxidant potential, while nitrite accumulation may adversely affect fruit firmness and polyphenol content.

The study revealed notable differences between blueberry cultivars regarding antioxidant properties and polyphenol content, especially anthocyanins, which are linked to enhanced bioactivity. Cultivars such as PT, EAR, and BRI demonstrated the highest antioxidant capacities, whereas AUR and SN showed lower levels. Somaclones, or plants derived from tissue culture, exhibited significant variation compared to their parent genotypes. Some somaclones, such as DK2 and SN2, demonstrated enhanced polyphenolic content and antioxidant activity, while others, like PT1, showed reductions. These findings underscore the unpredictable nature of somaclonal variation and its potential in breeding programs.

A negative correlation was found between the lightness of the fruit colour (CIE L value) and polyphenol content, with darker fruits possessing higher anthocyanin concentrations and stronger antioxidant properties. Environmental factors, such as sunlight exposure, temperature, and soil composition, were suggested to influence the biosynthesis of phenolic compounds, further underscoring the importance of genotype origin.

The study concluded that careful selection of genotypes, including consideration of the origin and somaclonal variation, is essential for breeding programs to improve the nutritional quality and health-promoting properties of highbush blueberries. While somaclonal variation offers opportunities to enhance polyphenol content and antioxidant activity, it also presents challenges, as unintended traits may arise. Thus, a thorough screening of somaclones is necessary to ensure their health benefits.

Additionally, the study found a significant positive correlation between total polyphenol content, particularly anthocyanins, and antioxidant activity, confirming that polyphenols are the primary contributors to the antioxidant properties of blueberries. The relationship between fruit firmness, sugar content, acidity, and bioactive compounds was also highlighted, suggesting that these characteristics may serve as indicators of both nutritional value and consumer appeal.

**Fig. 1:**
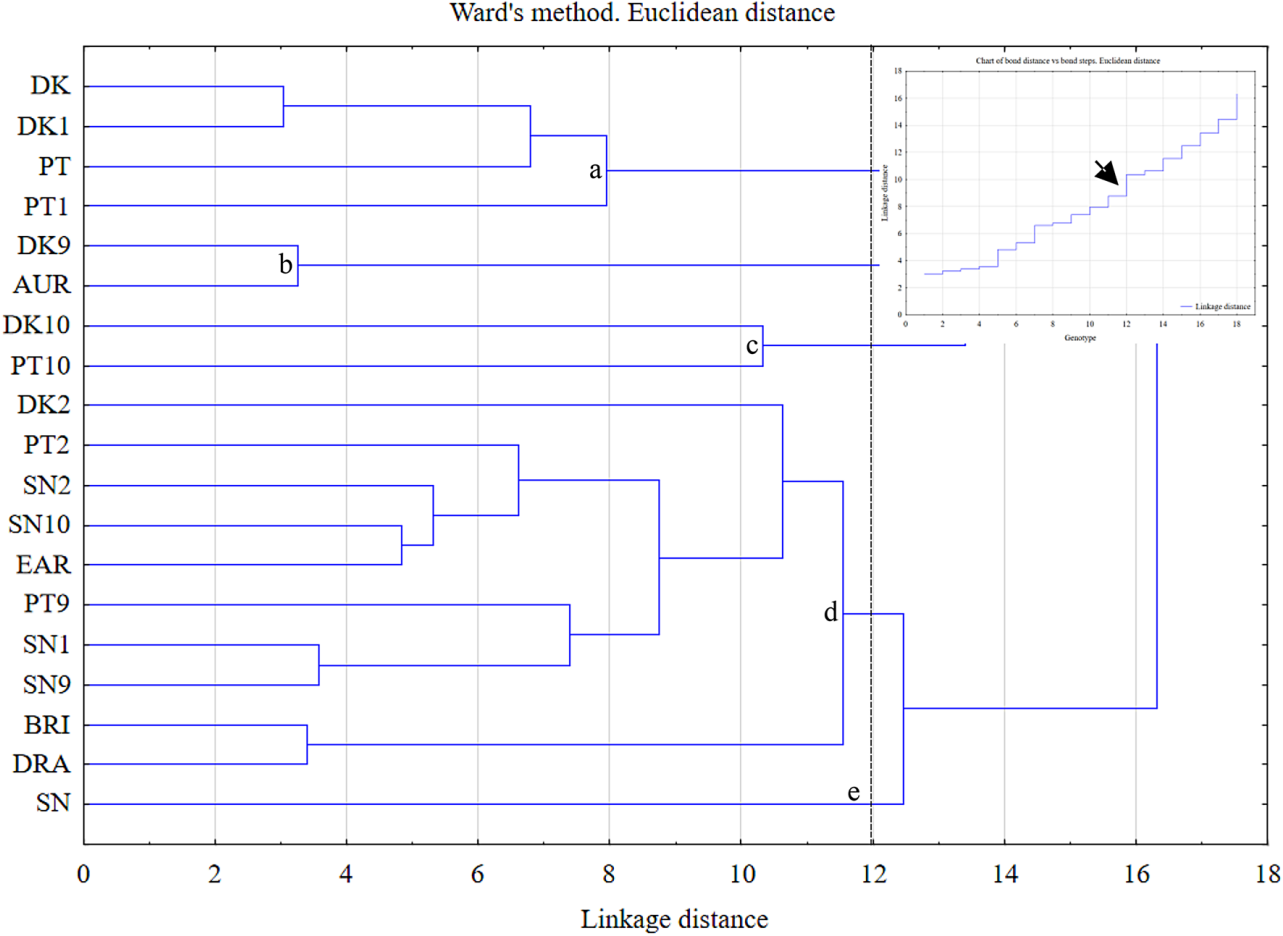
Dendrogram of cluster analyses for nineteen *Vaccinium corymbosum* L. genotypes based on the standardized values of the thirty-seven polyphenols used as variables, using Ward’s method. The vertical lines indicate the cut-off used to form the groups (a-e).

Agglomerative hierarchical clustering using Ward linkage was applied with Euclidean distances to demonstrate the relationships among the studied highbush blueberry genotypes. If we assume that the method’s essence is to cluster elements (genotypes) with minimal variance within a cluster, then genotypes with similar characteristics are grouped together. As a result, the genotypes are divided into distinct groups with similar traits. In this study, nineteen highbush blueberry genotypes were subjected to agglomerative clustering. The results of the analysis are presented as a dendrogram with five groups (a-e) representing the average contents of individual polyphenols from the following groups: phenolic acids, flavonols, flavan-3-ols, and anthocyanins (a-e) (Fig. 1).

Significant variability was confirmed when examining the polyphenol profiles among the studied cultivars (DK, SN, PT, AUR, BRI, DRA, and EAR). Notably, a high degree of similarity was observed between the BRI and DRA cultivars (group d) and their close relationship to the subgroup of group d represented by DK2, SN2, SN10, and EAR. An interesting observation was the assignment of DK, SN, and PT cultivars from different collections to different clusters (Fig. 1), as well as the distinct clustering of somaclones themselves. In relation to the somaclone and its originating genotype, attention is drawn to the grouping of DK-DK1 and PT-PT1 (a). They were very similar to each other. The grouping generated for the set of studied genotypes based on averages for polyphenol groups (from phenolic acids to anthocyanins, respectively) is presented in the form of dendrograms in Figure S1. The analysis of these results, as well as those derived from the dendrogram in Fig. 1, should be approached with caution to avoid overinterpretation. The dendrograms in Figure S1 show different groupings depending on the polyphenol group being analyzed. Interestingly, the DK-DK1 pair consistently appears in the same group (Figure S1).

**Fig. 2:**
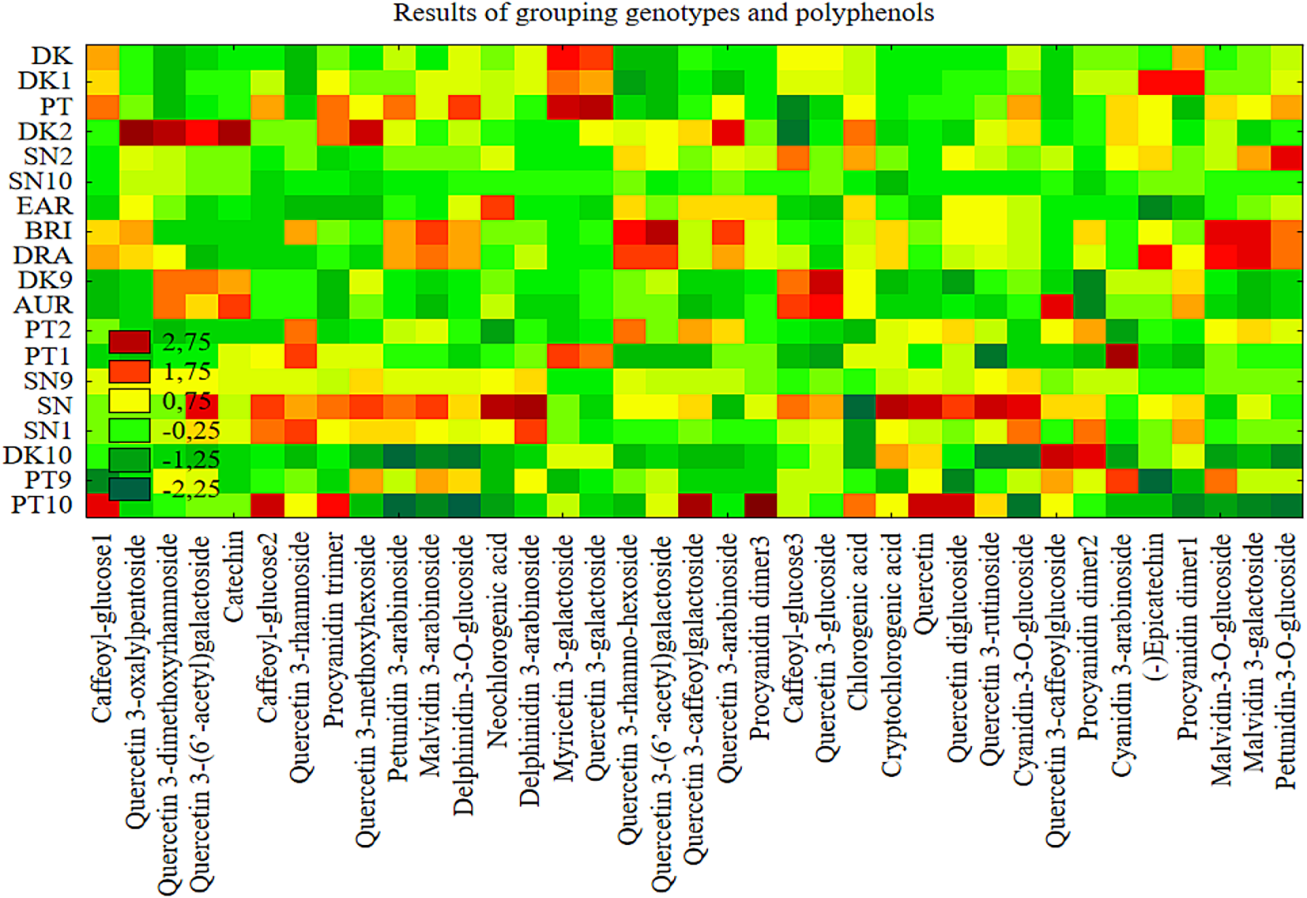
Results of clustering of *Vaccinium corrymbosum* L. genotypes and polyphenols. On a heatmap displaying standardized data, the color of each cell corresponds to the standardized value (z-score), i.e., the number of standard deviations by which an observation departs from the variable’s mean. Positive values (shades of red and orange) indicate levels above the mean (e.g., z ≈ + 1 is ~ 1 SD above the mean; z ≈ + 2 denotes markedly elevated values), values near zero (yellows) indicate agreement with the mean, and negative values (green to dark green) indicate levels below the mean (e.g., z ≈ − 1 is ~ 1 SD below the mean; z ≈ − 2 denotes markedly reduced values). In the legend shown, the scale ranges from approximately − 2.25 (dark green) to + 2.75 (dark red). It should be emphasized that these are dimensionless quantities and do not directly reflect concentration units, but rather the relative deviation from the mean.

Cluster analysis (as in Fig. 1 and Figure S1) was performed by grouping genotypes (cases) and variables (standardized averages for individual polyphenols) and presented in the form of heat maps (Fig. 2).

Genotypes with similar characteristics (i.e., based on the content of individual polyphenols) were classified. When analyzing the heat map, attention was drawn to the similar grouping of DK-DK1, as seen in Ward’s dendrogram (Fig. 1 and Figure S1). Notably, similar clustering of DK10 and PT10, as well as BRI and DRA (Fig. 2), was observed, along with significant variability in the occurrence of chlorogenic acid and malvidin-3-galactoside among the studied genotypes.

**Fig. 3:**
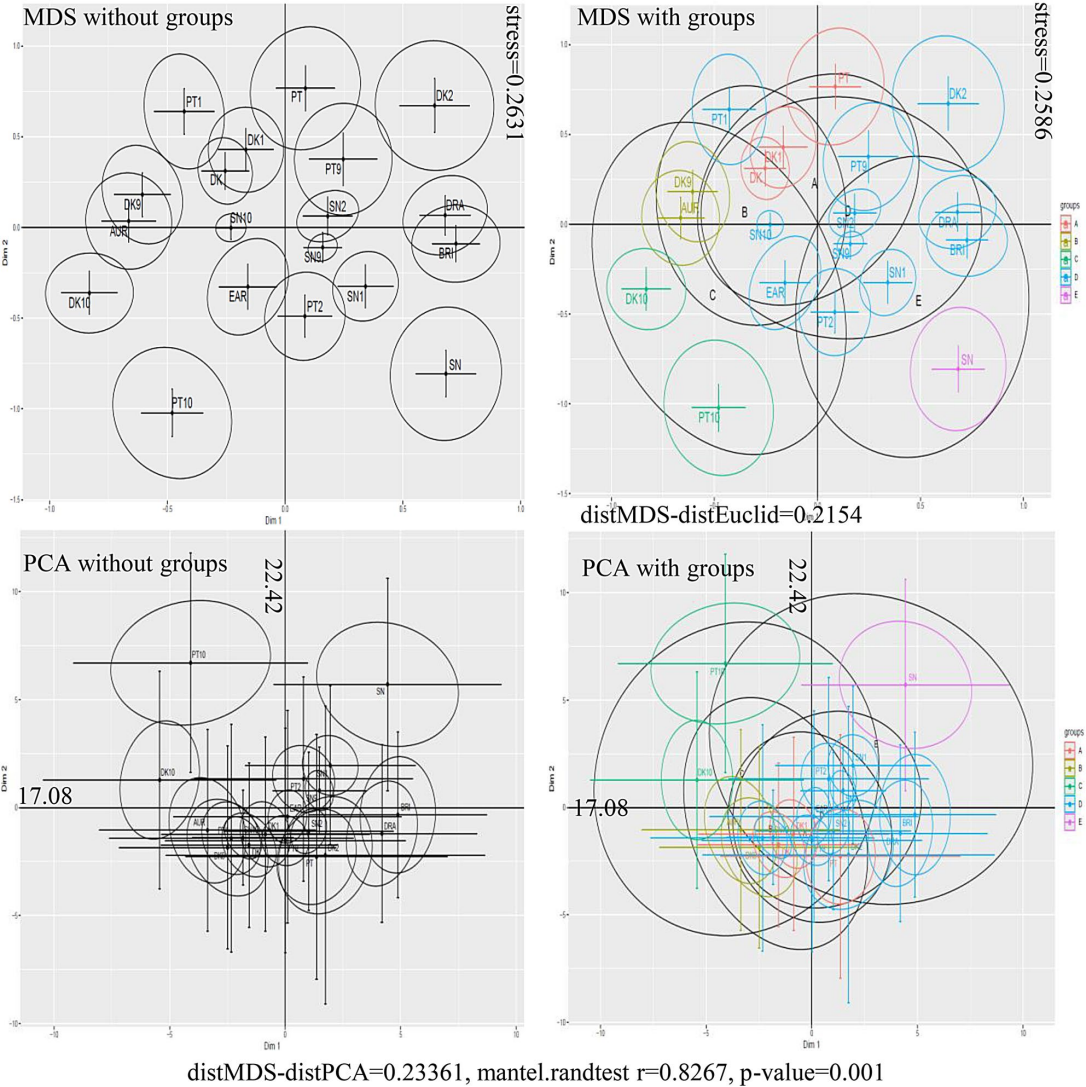
Comparison of MDS and PCA analysis results presented for thirty-seven polyphenols across 19 highbush blueberry genotypes. Legend. MDS without groups—distribution of means in 2D space without group membership; MDS, PCA distribution of means in 2D space according to genotype grouping as in Ward’s method. As presented in Smolik et al.’s^69^ paper, error bars are the square root of the stress statistics. Bootstrap shows the sensitivity of the spatial configuration of treatments to the missing random measurement in the dataset. The ellipses around the point (genotype) illustrate the covariance of 500 coordinates, assuming that the distribution of the resulting alternative coordinates of the point configurations is a two-dimensional normal distribution. Black ellipses represent the covariances of alternative coordinates belonging to the groups designated by Ward’s agglomerative method. Panels: (A) MDS without groups; (B) MDS with groups (Ward’s agglomerative clusters); (C) PCA without groups; (D) PCA with groups. Points denote genotype means in the 2D ordination. Crosshairs and ellipses summarize bootstrap dispersion (500 resamples); in the MDS panels, error bars correspond to √stress. Ellipses around each point depict the covariance of bootstrap coordinates assuming a bivariate normal distribution; black ellipses show covariances for clusters defined by Ward’s method.

Multi-Dimensional Scaling (MDS) and Principal Components Analysis (PCA), used in multivariate analyses, reduce dimensionality and improve the interpretability of study results^68^. They present multidimensional data in a lower-dimensional space while preserving the core characteristics of the data (Fig. 3 and Figure S2).

According to Albayrak Delialioğlu et al.^70^, the MDS method has also been applied in horticultural crops to establish trait associations and identify characteristics in apricots, as seen in cultivar groupings in apple^71^, mulberry^72^, and Brazil nut^69^. In this study, MDS was generated for 37 polyphenols (Fig. 3), as well as for specific polyphenol groups (Figure S3). Analysis of the MDS results highlighted variability in the distribution of points (genotypes) in the 2D space (groups a-e). Different clustering patterns were observed for cultivars, cultivars from different nurseries, somaclones, and the somaclones with their parent genotypes. Noteworthy is the proximity of DK and DK1, SN-SN1, DRA-BRI, and DK10-SN10 (Fig. 3).

In the PCA analysis, the first two principal components explained 22.42% and 17.08% of the total variance, respectively (Fig. 3). The values of PC1 and PC2 differentiate genotype groups from different collections. Group A samples are located in the negative portions of both PC1 and PC2, group B samples in the positive portions of both, group C samples in the negative part of PC1 and the positive part of PC2, group D samples in the positive part of PC1 and the negative part of PC2, and group E samples in the negative part of PC1 and the positive part of PC2. The distinct positioning of genotypes PT10, DK10, and SN was noted (Fig. 3).

In our study, we stated that for the research material, expanded by 18 new somaclones designated accordingly (DK3-8, SN3-9, PT3-8) and derived, similar to DK1-2, PT1-2, and SN1-2, from the DK, PT, and SN cultivars, additional studies were conducted^72,73^. Using RAPD (Random Amplified Polymorphic DNA), in addition to determining genetic diversity among the highbush blueberry cultivars, we demonstrated significant genetic variation within the same cultivars but from different collections. We also showed substantial genetic differences between selected somaclones and the highbush blueberry genotypes from which they were derived. Furthermore, using the Regularized Generalized Canonical Correlation Analysis (RGCCA) algorithm^74,75^, we identified a positive influence of specific RAPD loci on the levels of quercetin 3-galactoside, myricetin 3-galactoside, quercetin 3-(6’-acetyl)galactoside, and quercetin 3-rhamno-hexoside in the fruits of highbush blueberry.

### Summary of findings

This study revealed substantial variability in the physical, biochemical, and polyphenolic characteristics of highbush blueberry fruits depending on both genotypic origin and source of plant material. Among the cultivars studied, ‘Draper’ and ‘Brigitta’ consistently exhibited the highest total polyphenolic content and strongest antioxidant activity, highlighting the central role of genetic background.

Notably, identical cultivar names sourced from different nurseries showed significant differences in their phytochemical composition and bioactivity. Plants derived from German nurseries (e.g., DK, PT, SN) had elevated levels of anthocyanins and polyphenols compared to counterparts propagated in Poland, particularly from the Uroczysko nursery, which showed the lowest bioactive compound levels across all tested genotypes. These findings point to potential misidentification or somaclonal variation arising from prolonged *in vitro* micropropagation.

Somaclones such as SN2 demonstrated enhanced antioxidant capacity compared to parental lines, while PT1 showed reduced activity, underscoring the unpredictable outcomes of micropropagation. Although high genetic similarity was observed between DK and DK1 or SN and SN1, biochemical differences were still evident.

A strong negative correlation was observed between fruit lightness (CIE L*) and anthocyanin concentration—darker fruits contained more pigments and exhibited higher antioxidant potential. Similarly, firmer berries showed increased levels of ascorbic acid and stronger skin resistance, indicating that fruit texture may serve as a proxy for nutritional quality and postharvest durability.

Additional correlations indicated that berries with higher sugar and acidity levels tended to show stronger antioxidant activity, while the presence of nitrates and nitrites correlated with specific phenolic acids and antioxidant measures, suggesting an effect of cultivation practices on fruit biochemistry.

Importantly, total polyphenol content—especially anthocyanins—was positively associated with ABTS, DPPH, and FRAP antioxidant assays, reinforcing their role as primary contributors to blueberry health benefits. Lastly, environmental factors such as solar exposure, temperature, and soil characteristics appeared to modulate phenolic biosynthesis across genotypes, further emphasizing the combined influence of genotype and environment on fruit quality traits.

## Materials and methods

Plant research material of *Vaccinium corymbosum* L. originated from two experimental sites located in the West Pomeranian region of Poland: (1) a commercial farm “Blueamber” (BA), specialising in highbush blueberry cultivation, and (2) the Experimental Station of the Department of Horticulture, West Pomeranian University of Technology in Szczecin (WPUT) (Table 4).

**Table 4:** Characteristics of the plant material of *Vaccinium corymbosum* L. used in the experiment.

Genotypes and abbreviations		Donor	Origin of plant materials
Duke	DK	BA	imported (purchased) from Germany in the 90’s
Duke1	DK1		Duke’s somaclones are used to establish a plantation
Duke2	DK2		
Duke9	DK9	WPUT	imported (purchased) from nursery farm Ciepłucha (Poland)
Duke10	DK10		imported (purchased) from the ornamental shrubs nursery Uroczysko (Poland)
Patriot	PT	BA	plant material imported (purchased) from Germany in the 90’s
Patriot1	PT1		Patriot’s somaclones used to establish a plantation
Patriot2	PT2		
Patriot9	PT9	WPUT	imported (purchased) from nursery farm Ciepłucha (Poland)
Patriot10	PT10		imported (purchased) ornamental shrubs, nursery Uroczysko (Poland)
Sunrise	SN	BA	imported (purchased) from Germany in the 90’s
Sunrise1	SN1		Sunrise’s somaclones are used to establish a plantation
Sunrise2	SN2		
Sunrise9	SN9	WPUT	imported (purchased) from nursery farm Ciepłucha (Poland)
Sunrise10	SN10		imported (purchased) from the ornamental shrubs nursery Uroczysko (Poland)
Aurora	AUR		imported (purchased) from the ornamental shrubs nursery Uroczysko (Poland)
Brigitta	BRI		
Draper	DRA		
Earliblue	EAR		

BA – Blueamber; WUPT – West Pomeranian University of Technology in Szczecin, Poland.

The Blueamber farm, located approximately 20 km east of Szczecin, encompasses 170 ha of conventional and organic plantations established on acidic, high-organic peat soil extracted from a nearby peat bog. Selected genotypes were propagated via tissue culture in the farm’s *in vitro* laboratory, using modified MS or WPM media depending on the cultivar.

At the WPUT site, blueberry bushes were planted in the same type of peat substrate, transported directly from the Blueamber site, to ensure identical physicochemical properties of the growing medium across both locations. The substrate was classified as Baltic-type peat with a pH of 3.4–3.9, organic matter content of 55.3%, 5.8% organic carbon, and electrical conductivity of 0.27 mS/cm.

All experimental bushes were planted with the same spacing (1.2–2.3 m) and maintained under the same agronomic practices, including annual applications of KALISOP® (50 kg/ha K₂O), Patentkali® (50 kg/ha K₂O and 20 kg MgO), and ammonium sulfate (150 kg/ha). Bushes were pruned annually during the leafless period, removing approximately 25% of the oldest shoots. Paraffin oil was applied in autumn and spring to control pests. To prevent spring frost damage, protective sprays were used when air temperatures dropped below 0 °C.

Irrigation was performed using T-Tape drip lines (1 L/h emitters), and soil moisture was regularly monitored using contact tensiometers to maintain values within the optimal range of pF 1.8–2.1. These practices were identically implemented at both locations to minimise environmental variation.

The proximity of the two sites and the use of the same peat substrate ensured homogeneity of macro- and microenvironmental conditions, which allowed reliable detection of genotype- and propagation-related effects.

### Sampling of fruit

The fruits were harvested at full ripeness based on visual assessment of skin colour, in accordance with commercial standards: berries were considered ripe when they were uniformly blue-coloured and showed no trace of green or red hues near the stalk. In addition, harvest was guided by refractometric measurement of total soluble solids (TSS), which had to reach 13–15%. The fruit harvest was conducted manually and repeated four times per genotype from the same individual bushes to ensure homogeneity and maturity consistency.

From each harvest, three replicates of 250 g of randomly selected ripe berries were collected and immediately frozen in polyethylene bags at − 25 °C until chemical analysis.

### Colour measurement

Colour parameters were L* (L* = 100 means white; L* = 0 means black), a* (+ a* means redness; − a* means greenness), b* (+ b* means yellow; − b* means blue). Colour coordinates were determined in the CIE L*a*b* space for the 10 standard observers and the D 65 standard illuminant. CIE L*a*b* was measured using a spectrophotometer, KonicaMinolta CM-700d.

### General fruits parameters

Measurements were performed on juice obtained from fresh fruits immediately after harvest. For this purpose, 100 g of fruits were thoroughly homogenized in a ceramic mortar, and after 20 min, the fruit pulp was filtered using a vacuum pump with a synthetic filter insert. The resulting juice was used for the following analyses. The samples’ total soluble solid content (TSS) was measured at 20 °C with a digital refractometer (PAL-1, Atago, Japan) and expressed in %Brix. Titratable acidity (TA) was determined by titration of the aqueous extract with 0.1 N sodium hydroxide (NaOH) to pH 8.1 (Elmetron CX-732, Zabrze, Poland). The concentrations of nitrates (NO₃⁻), nitrites (NO₂⁻), and *L-*ascorbic acid in highbush blueberry fruits were determined using a colorimetric-reflectometric method with Merck Reflectoquant® test strips and the RQflex® 10 reader (Merck). Each measurement series was preceded by scanning the calibration code provided with the strips, and results were recorded in mg/L and recalculated to fresh fruit mass. Sample preparation involved a simple cold-water extraction: 10 g of homogenised fruit was diluted to 100 mL with ultrapure water, shaken, and clarified by filtration (0.45 µm). In cases of strong pigmentation or turbidity, additional dilutions were made to obtain a clear solution within the test range. For vitamin C, extracts were prepared and analysed immediately, kept cold and protected from light to prevent degradation. Measurements followed the manufacturer’s procedure: the reactive zone of the strip was dipped into the extract for 1–2 s, excess liquid was removed, and after the recommended reaction time (30–120 s), the strip was inserted into the reader for reflectometric detection. In cases of matrix interference (pigments, reducing agents), analyses were repeated after further filtration or dilution.

The antioxidant capacity of fruit extracts was determined using the ABTS•⁺, DPPH•, and FRAP assays.

For the ABTS assay, the radical cation (ABTS•⁺) was generated by oxidation of ABTS with potassium persulfate and diluted to an absorbance of 0.70 ± 0.02 at 734 nm. Fruit extracts were added, and the decrease in absorbance was measured after 6 min at 734 nm, following the procedure described by Lachowicz et al.^76^.

The DPPH assay was conducted with a methanolic DPPH solution adjusted to an absorbance of ~ 1.0 at 515 nm. The absorbance decrease was recorded after 30 min in the dark at room temperature, as outlined by Lachowicz et al.^76^.

The FRAP assay was performed according to Benzie & Devaki^77^. The FRAP reagent was freshly prepared by mixing acetate buffer (pH 3.6), 10 mM TPTZ solution in HCl, and 20 mM FeCl₃·6H₂O in a 10:1:1 ratio. The reaction mixture was incubated at 37 °C for 10 min, and absorbance was read at 593 nm.

For all assays, Trolox was used as a standard, and the results were expressed as millimoles Trolox equivalents per 100 g fresh matter (mmol TE/100 g fm). Measurements were performed in triplicate.

The firmness and puncture resistance of the skin were measured with a FirmTech2 apparatus (BioWorks, USA) on 100 randomly selected berries from three replicates; the values are expressed as gram force causing the fruit surface to bend 1 mm (G mm^–1^). Firmness was determined using a flat probe with a diameter of 3 cm, while skin puncture resistance was measured with a cylindrical probe of 4 mm in diameter, applied until the skin was ruptured and tissues were damaged. At this point, the software automatically stopped the measurement and recorded the force required to puncture the fruit skin.

### Extraction procedure and identification of polyphenol compounds

The fruits were frozen at − 25 °C, freeze-dried (24 h; FreeZone; Labconco Corporation, USA), and crushed (IKA A.11, Germany). The powders were kept in a refrigerator (− 25 °C) until analysis. All experiments in the manuscript were performed with relevant International Union for Conservation of Nature guidelines. Extraction for bioactive compounds: all samples (1 g) were extracted with 10 mL of a mixture containing UPLC-grade methanol (30%), acetic acid (1% of reagent), and distilled water (69%). The extraction was performed twice; the first incubation lasted 20 min at 25 °C under sonication (Sonic 6D, Polsonic, Warsaw, Poland), then the samples were stored at 4 °C for 24 h and then incubated again at 25 °C for 20 min in a sonication (Sonic 6D, Polsonic, Warsaw, Poland) and with occasional shaking. Next, the slurry was centrifuged at 19,000 *g* for 10 min, and the supernatant was filtered through a hydrophilic PTFE 0.20 μm membrane (Millex Samplicity Filter, Merck, Darmstadt, Germany) and used for analysis as described earlier^43^. Polyphenolic compounds were analysed using a UPLC-PDA-MS/MS Waters ACQUITY system (Waters, Milford, MA, USA), consisting of a binary pump manager, sample manager, column manager, PDA detector, and tandem quadrupole mass spectrometer (TQD) with electrospray ionization (ESI). The separation was carried out using a BEH C18 column (100 mm × 2.1 mm i.d., 1.7 µm, Waters) kept at 50 °C. For the anthocyanins investigation, the following solvent systems were applied: mobile phase A (2% formic acid in water, v/v) and mobile phase B (2% formic acid in 40% ACN in water, v/v). For other polyphenolic compounds, a lower formic acid concentration was used (0.1% v/v). The gradient program was set as follows: 0 min 5% B, from 0 to 8 min linear to 100% B, and from 8 to 9.5 min for washing and back to initial conditions. The total analysis time is 9.5 min. The injection volume of the samples was 5 µL (partial loop with needle overfill), and the flow rate was 0.35 mL/min. The following parameters were used for TQD: capillary voltage 3.5 kV; cone voltage 30 V in positive and negative mode; the source was kept at 250 °C, and desolvation temperature was 350 °C; cone gas flow 100 L/h; and desolvation gas flow 800 L/h. Argon was used as a collision gas at a 0.3 mL/min flow rate. The polyphenolic detection and identification were based on specific PDA spectra, mass-to-charge ratio, and fragment ions obtained after collision-induced dissociation (CID). The quantitative analysis was based on specific MS transitions in Multiple Reaction Monitoring (MRM) mode. Each polyphenolic compound’s MRM transitions, cone voltage, and collision energy were set manually with a dwell time of at least 25 ms. Before injection, samples were filtered through a 0.45 µm pore size membrane filter (Merck Millipore) and injected directly into the chromatographic column. The calibration curves were run at 360 nm for the standard myricetin 3-*O-*galactoside, quercetin 3-*O-*galactoside, quercetin 3-*O-*glucoside, at 320 nm for the standard of chlorogenic, caffeic, *p*-coumaric and sinapic acid, at 520 nm for the standard delphinidin 3-*O*-galactoside, peonidin 3-*O*-glucoside, cyanidin 3-*O*-galactoside and malvidin 3-*O*-glucoside and at 280 nm for the standard (−) epicatechin, (+)-catechin, procyanidins B_2_ and A. For each analyte, calibration curves (≥ 5–8 points, 0.05–5 mg/mL; R^2^ ≥ 0.9999) were constructed by plotting concentration versus peak area. Each curve was prepared in triplicate. From triplicate standard curves, the mean slope (S) and the standard deviation of the intercept (δ) were obtained, and compound-specific sensitivity was calculated as: LOD = 3.3 × (δ/S) and LOQ = 10 × (δ/S). Precision (intra- and inter-day) was evaluated as RSD (%) using QC solutions and matrix samples at ≥ 2 concentration levels; for all compounds, RSD < 3.5%. Significant variability was confirmed when examining the polyp. All determinations were performed in triplicate and expressed as mg/100 g fresh matter (fm). Waters MassLynx software v.4.1 was used for data acquisition and processing^77^.

### Statistical analysis

Part of the statistical analyses was performed using Statistica 13.3 (TIBCO, Poland). The data were analyzed using descriptive statistics and were subjected to one-factor ANOVA. Mean comparisons were performed using Tukey’s HSD test at *p* < 0.05. Prior to analysis, normality (Shapiro–Wilk test) was evaluated. Multiple comparisons were performed with Tukey’s HSD test. Statistical significance was set at α = 0.05 (two-tailed). Results are reported as mean ± SD. Using Statistica 13.3 (TIBCO, Poland), a hierarchical cluster analysis was performed on 19 genotypes (*V. corymbosum* L.), using as variables the concentrations of individual polyphenols determined for each genotype. For each compound, the mean of analytical replicates was calculated, yielding a 19 × p data matrix. Prior to analysis, the variables were standardized using the z-score method (mean = 0, standard deviation = 1) to balance the effects of differences in scale and variance. Based on the standardized data, a Euclidean distance matrix was computed, after which Ward’s agglomerative method was applied, minimizing the increase in the total within-cluster sum of squares at successive stages of merging observations. The results are presented as a dendrogram. The number of clusters was determined based on jumps in linkage heights (the “elbow” criterion). The study also includes a heat map of polyphenol concentrations ordered according to the dendrogram sequence, which facilitated the visual identification of chemical profiles characteristic of the identified clusters. MDS and PCA were performed in R^76^. The package SMACOF was used to perform MDS (Multi-Dimensional Scaling), stress, and bootstrap^78^, and APE^79^ to perform PCA (Principal Coordinates Analysis), and stress. Moreover, after standardizing the Nei distance matrices, we calculated the differences between the mean distances of the *Vaccinium* genotype pair plotted in the MDS and PCA plots, respectively, and the actual distance of the same pair on the Nei matrices^80–82^, 83-88]. Considering the MDS to PCA relation, as in the Smolik et al.^69^ paper, we have included them in the charts. Additional comments have been added to the figures below.

## Conclusions

This study demonstrates that both genotype and the origin of plant material decisively shape the polyphenolic composition, antioxidant potential, and physical quality of highbush blueberries, even when macro-environmental conditions and substrate are harmonized. Among the cultivars investigated, *Draper* and *Brigitta* consistently exhibited the highest total polyphenols—driven largely by anthocyanins and flavonols—and the strongest antioxidant activity, underscoring the primacy of genetic background for nutraceutical value. A consistent origin effect emerged for identically named cultivars sourced from different nurseries: German-origin plants (*DK, PT, SN*) tended to accumulate more anthocyanins and total polyphenols than their Polish counterparts (notably Uroczysko), translating into higher bioactivity and darker peel color. This finding points to propagation-related divergence, potential mislabeling, or somaclonal drift within commercial pipelines and has direct consequences for authenticity control, product standardization, and consumer value. Somaclonal variants further illustrated the bidirectional nature of micropropagation outcomes. *SN2* and *DK2* outperformed their parental lines in polyphenol content and antioxidant capacity, whereas *PT1* showed reductions—evidence that *in vitro* routes can yield both advantageous and disadvantageous shifts in secondary metabolism, warranting systematic screening before large-scale deployment. Correlation analyses bridged chemistry with quality traits: darker fruit (lower CIE L*) contained more anthocyanins and total polyphenols and showed higher DPPH/ABTS activity, while firmer berries also exhibited stronger skin resistance and higher *L-*ascorbic acid—traits relevant to postharvest robustness and perceived quality. These multivariate relationships support the use of simple physical metrics as practical proxies for bioactive potential. In parallel, our compound-level profiling highlighted notable chemical anchors—e.g., high chlorogenic acid across genotypes and unusually elevated catechin in *DK2*—that may inform targeted breeding and formulation strategies. Pattern-recognition approaches (Ward clustering, heatmaps, MDS, PCA) corroborated biologically meaningful groupings: *Brigitta* clustered closely with *Draper*; nursery-dependent separations were evident for *DK*, *PT*, and *SN*; and several somaclone–parent pairs occupied proximate ordinations, reflecting nuanced, trait-bounded divergence rather than wholesale reshaping of profiles. These findings validate polyphenol fingerprints as discriminants of genotype and origin.

Practical implications. For breeding and nursery practice, our results recommend: (i) molecular authentication and traceability for high-value planting material; (ii) routine phenotyping of somaclones to capture favorable chemotypes while avoiding unintended trade-offs; and (iii) source-aware procurement for growers and processors targeting functional foods, where consistent anthocyanin/phenolic levels are pivotal to label claims and health value.

Limitations and future work. While our design minimized macro-environmental noise, the study is single-season and site-constrained; future efforts should extend to multi-year, multi-location validations, integrate genomic markers and authentication assays, and quantify postharvest stability and processing resilience of key phenolics to bridge agricultural selection with product performance.

In sum, genotype × origin interactions, compounded by somaclonal dynamics, are central levers of blueberry bioactivity and quality. Aligning breeding, nursery propagation, and sourcing decisions with these determinants will accelerate the delivery of reliably bioactive, consumer-preferred fruit and ingredients to fresh and nutraceutical markets.

## Supplementary Information

Supplementary Information.

## Data Availability

The datasets used and analysed during the current study are available from the corresponding author on reasonable request.
