# Supplementary material for: Genotype and environment synergistically determine the polyphenolic composition and functional activity of highbush blueberries
Source: Sci Rep. 2025 Nov 13;15:39895. doi: 10.1038/s41598-025-23613-8 (PMC12615837; doi:10.1038/s41598-025-23613-8)
Supplement: Supplementary file 1 — Supplementary Information. [file 41598_2025_23613_MOESM1_ESM.docx]

**Supplementary Table 1A.** Single polyphenol compound concentrations in different *Vaccinium corymbosum* L. genotypes (mg/100 g fresh matter)

| Genotype |  | Caffeoyl-glucose |  | Caffeoyl-glucose |  | Caffeoyl-glucose |  | neochlorogenic acid |  | chlorogenic acid |  | Cryptochlorogenic acid |  |
| --- | --- | --- | --- | --- | --- | --- | --- | --- | --- | --- | --- | --- | --- |
| DK | Phenolic acid | 5.04±0.07 | ^c^ | 0.36±0.01 | ^k^ | 1.72±0.02 | ^b^ | 1.72±0.01 | ^de^ | 132.53±0.55 | ^bcd^ | 0.67±0.01 | ^l^ |
| DK1 |  | 4.93±0.02 | ^d^ | 0.75±0.01 | ^g^ | 1.69±0.01 | ^bc^ | 1.90±0.01 | ^d^ | 135.16±1.07 | ^ad^ | 0.76±0.01 | ^k^ |
| DK2 |  | 3.14±0.03 | ^j^ | 0.62±0.02 | ^h^ | 0.32±0.01 | ^h^ | 1.43±0.02 | ^f^ | 157.97±1.49 | ^a^ | 0.36±0.01 | ^m^ |
| DK9 |  | 2.23±0.02 | ^n^ | 0.50±0.02 | ^i^ | 2.22±0.01 | ^a^ | 1.75±0.01 | ^de^ | 145.88±1.16 | ^abc^ | 0.32±0.01 | ^m^ |
| DK10 |  | 3.30±0.03 | ^i^ | 0.40±0.01 | ^j^ | 1.21±0.00 | ^de^ | 0.83±0.02 | ^g^ | 102.61±0.65 | ^ef^ | 2.62±0.03 | ^b^ |
| PT |  | 5.49±0.02 | ^b^ | 1.30±0.01 | ^d^ | 0.42±0.02 | ^h^ | 1.96±0.02 | ^d^ | 144.57±1.56 | ^abc^ | 0.76±0.01 | ^k^ |
| PT1 |  | 2.54±0.01 | ^l^ | 1.14±0.02 | ^e^ | 0.73±0.42 | ^g^ | 1.56±0.42 | ^ef^ | 136.80±1.11 | ^ad^ | 1.65±0.01 | ^g^ |
| PT2 |  | 3.62±0.04 | ^g^ | 0.32±0.01 | ^lm^ | 1.07±0.03 | ^ef^ | 0.52±0.02 | ^h^ | 53.08±42.02 | ^g^ | 1.79±0.02 | ^f^ |
| PT9 |  | 1.87±0.02 | ^o^ | 0.54±0.01 | ^i^ | 1.43±0.02 | ^cd^ | 0.61±0.02 | ^gh^ | 123.62±0.36 | ^ce^ | 0.25±0.01 | ^n^ |
| PT10 |  | 6.17±0.03 | ^a^ | 2.08±0.03 | ^a^ | 1.54±0.03 | ^bc^ | 0.40±0.01 | ^h^ | 156.98±0.98 | ^ab^ | 1.93±0.01 | ^e^ |
| SN |  | 3.70±0.01 | ^g^ | 1.67±0.02 | ^b^ | 2.25±0.03 | ^a^ | 4.46±0.04 | ^a^ | 83.02±0.17 | ^f^ | 4.05±0.06 | ^a^ |
| SN1 |  | 3.52±0.03 | ^h^ | 1.53±0.02 | ^c^ | 1.51±0.02 | ^bc^ | 2.42±0.02 | ^c^ | 99.37±0.59 | ^ef^ | 2.15±0.01 | ^d^ |
| SN2 |  | 3.09±0.02 | ^jk^ | 0.35±0.02 | ^kl^ | 2.18±0.03 | ^a^ | 2.28±0.03 | ^c^ | 152.55±0.49 | ^ab^ | 1.16±0.01 | ^i^ |
| SN9 |  | 4.29±0.03 | ^f^ | 0.95±0.02 | ^f^ | 1.22±0.01 | ^de^ | 2.43±0.02 | ^c^ | 120.74±0.59 | ^ce^ | 1.51±0.02 | ^h^ |
| SN10 |  | 3.02±0.03 | ^k^ | 0.34±0.02 | ^kl^ | 1.24±0.03 | ^de^ | 1.55±0.01 | ^ef^ | 115.58±0.96 | ^de^ | 0.10±0.00 | ^o^ |
| AUR |  | 2.37±0.04 | ^m^ | 0.54±0.01 | ^i^ | 2.35±0.03 | ^a^ | 1.96±0.01 | ^d^ | 141.06±0.69 | ^ac^ | 0.36±0.01 | ^m^ |
| BRI |  | 4.64±0.09 | ^e^ | 0.28±0.01 | ^m^ | 1.19±0.01 | ^df^ | 1.77±0.02 | ^de^ | 134.45±1.13 | ^ad^ | 2.41±0.03 | ^c^ |
| DRA |  | 5.04±0.07 | ^c^ | 0.28±0.01 | ^m^ | 1.55±0.02 | ^bc^ | 1.52±0.01 | ^ef^ | 140.55±0.33 | ^ad^ | 2.38±0.03 | ^c^ |
| EAR |  | 2.51±0.01 | ^l^ | 0.31±0.01 | ^lm^ | 0.94±0.01 | ^fg^ | 3.33±0.02 | ^b^ | 149.1±0.92 | ^ab^ | 0.93±0.03 | ^j^ |
| Mean±SD |  | 3.71±1.20 | | 0.75±0.50 | | 1.41±0.60 | | 1.81±0.90 | | 127.7±27.90 | | 1.38±1.00 | |
| Min-Max |  | 1.8-6.2 | | 0.26-2.1 | | 0.18-2.4 | | 0.38-4.5 | | 10-160 | | 0.1-4.1 | |
| CV% |  | 32.4 |  | 71.0 |  | 41.2 |  | 52.5 |  | 21.8 |  | 74.2 |  |

| Genotype |  | Myricetin 3-galactoside |  | Quercetin diglucoside |  | Quercetin 3-rhamno-hexoside |  | Quercetin 3-rutinoside |  | Quercetin 3-galactoside |  | Quercetin 3-methoxyhexoside |  | Quercetin 3-glucoside |  | Quercetin 3-arabinoside |  |
| --- | --- | --- | --- | --- | --- | --- | --- | --- | --- | --- | --- | --- | --- | --- | --- | --- | --- |
| DK | Flavonols | 5.71±0.02 | ^b^ | 0.32±0.01 | ^h^ | 0.66±0.01 | ^n^ | 1.07±0.02 | ^k^ | 7.89±0.06 | ^b^ | 1.85±0.03 | ^i^ | 1.93±0.02 | ^d^ | 0.7±0.42 | ^kl^ |
| DK1 |  | 5.13±0.03 | ^d^ | 0.40±0.00 | ^g^ | 0.21±0.01 | ^p^ | 1.16±0.01 | ^j^ | 7.06±0.06 | ^d^ | 2.11±0.01 | ^g^ | 1.83±0.02 | ^e^ | 0.19±0.01 | ^n^ |
| DK2 |  | 1.84±0.01 | ^kl^ | 0.23±0.01 | ^i^ | 2.55±0.02 | ^g^ | 1.69±0.02 | ^e^ | 5.73±0.04 | ^e^ | 3.21±0.02 | ^a^ | 0.98±0.03 | ^k^ | 3.73±0.02 | ^a^ |
| DK9 |  | 1.77±0.03 | ^l^ | 0.12±0.01 | ^j^ | 2.06±0.03 | ^i^ | 1.25±0.02 | ^i^ | 3.78±0.03 | ^g^ | 2.32±0.02 | ^f^ | 3.34±0.03 | ^a^ | 0.47±0.01 | ^lm^ |
| DK10 |  | 3.54±0.01 | ^e^ | 0.35±0.01 | ^h^ | 0.46±0.02 | ^o^ | 0.36±0.00 | ^o^ | 5.57±0.02 | ^f^ | 1.43±0.01 | ^m^ | 1.51±0.02 | ^g^ | 0.13±0.00 | ^n^ |
| PT |  | 6.52±0.03 | ^a^ | 0.39±0.01 | ^g^ | 0.92±0.01 | ^m^ | 1.42±0.01 | ^h^ | 10.57±0.19 | ^a^ | 2.39±0.03 | ^e^ | 0.83±0.02 | ^l^ | 1.99±0.03 | ^ef^ |
| PT1 |  | 5.47±0.03 | ^c^ | 0.54±0.02 | ^e^ | 0.61±0.02 | ^n^ | 0.42±0.02 | ^m^ | 7.38±0.03 | ^c^ | 2.27±0.02 | ^f^ | 0.37±0.02 | ^o^ | 1.28±0.01 | ^hi^ |
| PT2 |  | 1.57±0.01 | ^m^ | 0.68±0.01 | ^c^ | 3.87±0.02 | ^c^ | 1.51±0.01 | ^g^ | 2.83±0.02 | ^k^ | 1.81±0.03 | ^i^ | 0.76±0.02 | ^m^ | 2.38±0.02 | ^d^ |
| PT9 |  | 1.26±0.01 | ^n^ | 0.08±0.01 | ^k^ | 2.06±0.01 | ^i^ | 1.26±0.01 | ^i^ | 3.26±0.02 | ^hi^ | 2.62±0.02 | ^c^ | 1.68±0.02 | ^f^ | 0.52±0.02 | ^lm^ |
| PT10 |  | 3.05±0.05 | ^f^ | 1.12±0.01 | ^a^ | 0.49±0.01 | ^o^ | 1.89±0.02 | ^b^ | 2.41±0.02 | ^m^ | 1.57±0.02 | ^l^ | 1.27±0.02 | ^i^ | 0.81±0.02 | ^jk^ |
| SN |  | 2.83±0.02 | ^g^ | 0.90±0.01 | ^b^ | 3.03±0.02 | ^f^ | 2.80±0.01 | ^a^ | 2.69±0.01 | ^l^ | 2.86±0.01 | ^b^ | 2.42±0.01 | ^c^ | 0.19±0.00 | ^n^ |
| SN1 |  | 2.48±0.03 | ^h^ | 0.48±0.01 | ^f^ | 1.63±0.02 | ^l^ | 1.70±0.02 | ^e^ | 2.97±0.03 | ^jk^ | 2.53±0.02 | ^d^ | 1.84±0.04 | ^e^ | 1.04±0.02 | ^ij^ |
| SN2 |  | 1.84±0.01 | ^kl^ | 0.65±0.01 | ^cd^ | 3.11±0.01 | ^e^ | 1.52±0.03 | ^g^ | 3.08±0.02 | ^j^ | 1.84±0.02 | ^i^ | 1.49±0.02 | ^g^ | 1.76±0.03 | ^fg^ |
| SN9 |  | 2.08±0.03 | ^j^ | 0.49±0.03 | ^f^ | 2.43±0.03 | ^h^ | 1.84±0.01 | ^c^ | 3.34±0.03 | ^hi^ | 2.56±0.03 | ^d^ | 1.46±0.01 | ^g^ | 1.53±0.01 | ^gh^ |
| SN10 |  | 2.16±0.01 | ^i^ | 0.32±0.01 | ^h^ | 1.94±0.02 | ^j^ | 1.14±0.01 | ^j^ | 3.74±0.03 | ^g^ | 1.71±0.02 | ^j^ | 1.38±0.02 | ^h^ | 1.24±0.02 | ^i^ |
| AUR |  | 1.54±0.01 | ^m^ | 0.33±0.02 | ^h^ | 1.79±0.02 | ^k^ | 0.93±0.01 | ^l^ | 3.40±0.02 | ^h^ | 2.08±0.03 | ^g^ | 3.06±0.04 | ^b^ | 0.33±0.02 | ^mn^ |
| BRI |  | 2.08±0.03 | ^j^ | 0.62±0.01 | ^d^ | 4.40±0.04 | ^a^ | 1.75±0.02 | ^d^ | 3.70±0.05 | ^g^ | 1.93±0.02 | ^h^ | 1.05±0.04 | ^j^ | 2.97±0.02 | ^b^ |
| DRA |  | 1.88±0.03 | ^k^ | 0.55±0.02 | ^e^ | 4.09±0.02 | ^b^ | 1.61±0.02 | ^f^ | 3.25±0.03 | ^i^ | 1.76±0.01 | ^j^ | 0.89±0.02 | ^l^ | 2.64±0.04 | ^c^ |
| EAR |  | 2.08±0.01 | ^j^ | 0.65±0.02 | ^cd^ | 3.38±0.03 | ^d^ | 1.84±0.03 | ^c^ | 2.56±0.03 | ^l^ | 1.66±0.01 | ^k^ | 0.55±0 | ^n^ | 2.21±0.01 | ^de^ |
| Mean±SD |  | 2.89±1.58 | | 0.49±0.25 | | 2.09±1.29 | | 1.43±0.54 | | 4.49±2.21 | | 2.13±0.47 | | 1.51±0.77 | | 1.37±1.03 | |
| Min-Max |  | 1.32-6.61 | | 0.07-1.13 | | 0.22-4.44 | | 0.41-2.81 | | 2.39-10.77 | | 1.41-3.24 | | 0.35-3.37 | | 0.12-3.77 | |
| CV% |  | 54.6 |  | 51.7 |  | 61.6 |  | 37.8 |  | 49.3 |  | 21.8 |  | 51.2 |  | 75.3 |  |

| Genotype |  | Quercetin 3-caffeoylgalactoside |  | Quercetin 3-caffeoylglucoside |  | Quercetin 3-oxalylpentoside |  | Quercetin 3-rhamnoside |  | Quercetin 3-dimethoxyrhamnoside |  | Quercetin 3-(6’-acetyl)galactoside |  | Quercetin 3-(6’-acetyl)galactoside |  | Quercetin |  |
| --- | --- | --- | --- | --- | --- | --- | --- | --- | --- | --- | --- | --- | --- | --- | --- | --- | --- |
| DK | Flavonols | 0.31±0.01 | ^i^ | 0.12±0.00 | ^lm^ | 1.51±0.01 | ^h^ | 0.29±0.01 | ^lm^ | 0.03±0.00 | ^k^ | 0.05±0.00 | ^jk^ | 0.01±0.00 | ^i^ | 0.06±0.00 | ^ij^ |
| DK1 |  | 0.26±0.01 | ^j^ | 0.13±0.01 | ^lm^ | 1.61±0.02 | ^h^ | 0.26±0.01 | ^mn^ | 0.04±0.01 | ^k^ | 0.10±0.00 | ^fg^ | 0.01±0.01 | ^i^ | 0.06±0.01 | ^i^ |
| DK2 |  | 0.59±0.01 | ^d^ | 0.31±0.02 | ^k^ | 8.45±0.05 | ^a^ | 0.65±0.01 | ^g^ | 2.22±0.02 | ^a^ | 0.29±0.01 | ^ab^ | 0.32±0.01 | ^cd^ | 0.03±0.00 | ^kl^ |
| DK9 |  | 0.23±0.01 | ^j^ | 0.10±0.00 | ^m^ | 0.36±0.01 | ^ij^ | 0.56±0.01 | ^i^ | 1.48±0.02 | ^b^ | 0.24±0.01 | ^b^ | 0.23±0.00 | ^ef^ | 0.01±0.00 | ^m^ |
| DK10 |  | 0.15±0.00 | ^k^ | 5.58±0.02 | ^a^ | 1.46±0.02 | ^h^ | 0.25±0.01 | ^n^ | 0.13±0.01 | ^j^ | 0.04±0.00 | ^k^ | 0.08±0.01 | ^h^ | 0.29±0.01 | ^c^ |
| PT |  | 0.34±0.01 | ^h^ | 0.17±0.01 | ^l^ | 1.93±0.01 | ^g^ | 0.32±0.00 | ^k^ | 0.04±0.00 | ^k^ | 0.07±0.00 | ^hi^ | 0.01±0.00 | ^i^ | 0.09±0.00 | ^h^ |
| PT1 |  | 0.18±0.01 | ^k^ | 0.13±0.01 | ^lm^ | 0.06±0.00 | ^j^ | 1.21±0.01 | ^b^ | 0.27±0.05 | ^h^ | 0.08±0.00 | ^gh^ | 0.01±0.00 | ^i^ | 0.06±0.01 | ^i^ |
| PT2 |  | 0.68±0.02 | ^b^ | 2.66±0.02 | ^e^ | 0.33±0.01 | ^j^ | 1.16±0.01 | ^c^ | 0.04±0.01 | ^k^ | 0.06±0.00 | ^ij^ | 0.20±0.00 | ^fg^ | 0.23±0.00 | ^d^ |
| PT9 |  | 0.23±0.01 | ^j^ | 3.55±0.03 | ^c^ | 0.32±0.01 | ^j^ | 0.61±0.01 | ^h^ | 1.08±0.02 | ^c^ | 0.15±0.01 | ^e^ | 0.25±0.00 | ^e^ | 0.23±0.01 | ^d^ |
| PT10 |  | 1.03±0.02 | ^a^ | 2.68±0.02 | ^e^ | 0.65±0.01 | ^i^ | 0.84±0.01 | ^e^ | 0.39±0.01 | ^g^ | 0.10±0.01 | ^f^ | 0.27±0.01 | ^de^ | 0.49±0.01 | ^b^ |
| SN |  | 0.61±0.01 | ^cd^ | 3.01±0.02 | ^d^ | 1.97±0.02 | ^g^ | 1.06±0.01 | ^d^ | 0.62±0.01 | ^e^ | 0.30±0.00 | ^a^ | 0.34±0.00 | ^c^ | 0.52±0.01 | ^a^ |
| SN1 |  | 0.41±0.01 | ^rg^ | 0.89±0.01 | ^h^ | 2.69±0.02 | ^ef^ | 1.24±0.02 | ^a^ | 0.71±0.00 | ^d^ | 0.19±0.01 | ^cd^ | 0.17±0.01 | ^g^ | 0.15±0.00 | ^f^ |
| SN2 |  | 0.39±0.01 | ^g^ | 1.93±0.06 | ^f^ | 2.93±0.07 | ^e^ | 0.61±0.01 | ^h^ | 0.68±0.01 | ^d^ | 0.10±0.01 | ^f^ | 0.34±0.01 | ^c^ | 0.05±0.00 | ^ij^ |
| SN9 |  | 0.46±0.01 | ^e^ | 1.53±0.04 | ^g^ | 3.30±0.03 | ^d^ | 0.74±0.01 | ^f^ | 1.08±0.05 | ^c^ | 0.18±0.01 | ^d^ | 0.25±0.00 | ^e^ | 0.18±0.00 | ^e^ |
| SN10 |  | 0.32±0.01 | ^hi^ | 0.70±0.01 | ^i^ | 2.48±0.02 | ^f^ | 0.41±0.01 | ^j^ | 0.72±0.02 | ^d^ | 0.10±0.01 | ^f^ | 0.09±0.09 | ^h^ | 0.04±0.00 | ^jk^ |
| AUR |  | 0.18±0.00 | ^k^ | 5.08±0.03 | ^b^ | 0.35±0.02 | ^j^ | 0.54±0.01 | ^i^ | 1.44±0.02 | ^b^ | 0.20±0.01 | ^c^ | 0.22±0.00 | ^eg^ | 0.01±0.00 | ^m^ |
| BRI |  | 0.44±0.01 | ^ef^ | 0.37±0.01 | ^j^ | 4.48±0.49 | ^b^ | 1.07±0.02 | ^d^ | 0.18±0.01 | ^i^ | 0.05±0.00 | ^jk^ | 0.65±0.01 | ^a^ | 0.12±0.00 | ^g^ |
| DRA |  | 0.42±0.03 | ^fg^ | 0.35±0.02 | ^jk^ | 3.81±0.05 | ^c^ | 0.31±0.01 | ^kl^ | 1.09±0.02 | ^c^ | 0.02±0.00 | ^l^ | 0.5±0.00 | ^b^ | 0.07±0.00 | ^h^ |
| EAR |  | 0.63±0.01 | ^c^ | 0.38±0.01 | ^j^ | 3.38±0.02 | ^d^ | 0.30±0.01 | ^kl^ | 0.56±0.01 | ^f^ | 0.04±0.00 | ^k^ | 0.22±0.00 | ^eg^ | 0.02±0.00 | ^lm^ |
| Mean±SD |  | 0.42±0.22 | | 1.56±1.7 | | 2.22±1.97 | | 0.66±0.34 | | 0.67±0.59 | | 0.13±0.08 | | 0.22±0.17 | | 0.14±0.15 | |
| Min-Max |  | 0.15-1.06 | | 0.11-5.61 | | 0.06-8.47 | | 0.24-1.27 | | 0.03-2.23 | | 0.02-0.31 | | 0.00-0.67 | | 0.01-0.53 | |
| CV% |  | 51.8 |  | 109.2 |  | 88.9 |  | 52.0 |  | 87.7 |  | 66.1 |  | 76.1 |  | 103.6 |  |

| Genotype |  | Procyanidin dimer |  | Procyanidin dimer |  | Catechin |  | Procyanidin dimer |  | (-)Epicatechin |  | Procyanidin trimer |  |
| --- | --- | --- | --- | --- | --- | --- | --- | --- | --- | --- | --- | --- | --- |
| DK | Flavan-3-ols | 8.81±0.02 | ^b^ | 3.75±0.01 | ^j^ | 8.41±0.01 | ^k^ | 1.76±0.03 | ^o^ | 4.36±0.02 | ^i^ | 1.21±0.01 | ^g^ |
| DK1 |  | 10.4±0.01 | ^a^ | 4.18±0.03 | ^i^ | 8.84±0.05 | ^j^ | 2.28±0.03 | ^m^ | 8.23±0.02 | ^a^ | 1.91±0.01 | ^d^ |
| DK2 |  | 4.13±0.02 | ^k^ | 3.54±0.07 | ^k^ | 37.47±0.53 | ^a^ | 3.62±0.03 | ^h^ | 6.37±0.04 | ^c^ | 2.41±0.03 | ^c^ |
| DK9 |  | 7.92±0.02 | ^d^ | 1.43±0.01 | ^s^ | 22.50±0.04 | ^c^ | 2.31±0.01 | ^m^ | 5.44±0.05 | ^f^ | 0.53±0.02 | ^l^ |
| DK10 |  | 6.71±0.02 | ^g^ | 7.52±0.03 | ^a^ | 4.53±0.01 | ^n^ | 1.20±0.01 | ^p^ | 4.98±0.03 | ^g^ | 0.96±0.01 | ^i^ |
| PT |  | 3.26±0.01 | ^m^ | 3.55±0.03 | ^k^ | 8.79±0.05 | ^jk^ | 2.19±0.01 | ^n^ | 6.10±0.01 | ^e^ | 2.47±0.02 | ^b^ |
| PT1 |  | 3.39±0.01 | ^l^ | 2.43±0.04 | ^p^ | 16.30±0.22 | ^d^ | 3.41±0.01 | ^i^ | 3.44±0.02 | ^k^ | 1.67±0.02 | ^e^ |
| PT2 |  | 4.17±0.01 | ^k^ | 5.97±0.03 | ^c^ | 5.37±0.02 | ^m^ | 2.78±0.03 | ^k^ | 4.41±0.01 | ^i^ | 0.76±0.02 | ^j^ |
| PT9 |  | 3.08±0.03 | ^o^ | 4.56±0.03 | ^g^ | 5.25±0.03 | ^m^ | 1.21±0.01 | ^p^ | 1.42±0.02 | ^n^ | 0.72±0.01 | ^j^ |
| PT10 |  | 2.08±0.02 | ^p^ | 3.43±0.02 | ^l^ | 11.12±0.03 | ^i^ | 17.47±0.05 | ^a^ | 3.16±0.01 | ^l^ | 2.85±0.03 | ^a^ |
| SN |  | 7.72±0.01 | ^e^ | 5.54±0.04 | ^d^ | 13.21±0.06 | ^f^ | 5.26±0.03 | ^e^ | 6.31±0.07 | ^cd^ | 2.45±0.03 | ^bc^ |
| SN1 |  | 8.51±0.04 | ^c^ | 6.39±0.02 | ^b^ | 16.56±0.19 | ^d^ | 3.14±0.05 | ^j^ | 4.94±0.01 | ^g^ | 1.94±0.03 | ^d^ |
| SN2 |  | 5.56±0.02 | ^i^ | 3.13±0.02 | ^m^ | 12.23±0.08 | ^g^ | 4.38±0.04 | ^f^ | 6.51±0.08 | ^b^ | 0.76±0.02 | ^j^ |
| SN9 |  | 5.2±0.01 | ^j^ | 4.44±0.03 | ^h^ | 15.78±0.25 | ^e^ | 3.85±0.08 | ^g^ | 4.22±0.04 | ^j^ | 1.58±0.01 | ^f^ |
| SN10 |  | 5.63±0.02 | ^h^ | 2.79±0.02 | ^o^ | 11.72±0.12 | ^h^ | 3.16±0.03 | ^j^ | 4.66±0.04 | ^h^ | 0.91±0.02 | ^i^ |
| AUR |  | 8.54±0.02 | ^c^ | 1.61±0.02 | ^r^ | 25.49±0.26 | ^b^ | 2.56±0.02 | ^l^ | 5.02±0.04 | ^g^ | 0.64±0.02 | ^k^ |
| BRI |  | 5.63±0.02 | ^h^ | 5.39±0.02 | ^e^ | 5.53±0.03 | ^m^ | 5.74±0.02 | ^d^ | 6.22±0.02 | ^d^ | 1.22±0.01 | ^g^ |
| DRA |  | 7.58±0.05 | ^f^ | 4.88±0.03 | ^f^ | 4.26±0.03 | ^n^ | 5.84±0.03 | ^c^ | 8.18±0.02 | ^a^ | 1.05±0.02 | ^h^ |
| EAR |  | 3.20±0.01 | ^n^ | 2.93±0.02 | ^n^ | 7.14±0.03 | ^l^ | 7.02±0.04 | ^b^ | 2.23±0.06 | ^m^ | 0.49±0.01 | ^l^ |
| Mean±SD |  | 5.87±2.35 | | 4.08±1.58 | | 12.66±8.30 | | 4.17±3.53 | | 5.06±1.74 | | 1.40±0.73 | |
| Min-Max |  | 2.06-10.41 | | 1.41-7.58 | | 4.28-38.22 | | 1.18-17.52 | | 1.39-8.25 | | 0.48-2.88 | |
| CV% |  | 40.0 |  | 38.6 |  | 65.6 |  | 84.6 |  | 34.5 |  | 52.6 |  |

| Genotype |  | Cyanidin-3-O-glucoside |  | Delphinidin-3-O-glucoside |  | Malvidin-3-O-glucoside |  | Petunidin-3-O-glucoside |  | Delphinidin 3-arabinoside |  | Cyanidin 3-arabinoside |  | petunidin 3-arabinoside |  | malvidin 3-galactoside |  | malvidin 3-arabinoside |  |
| --- | --- | --- | --- | --- | --- | --- | --- | --- | --- | --- | --- | --- | --- | --- | --- | --- | --- | --- | --- |
| DK | Anthocyanins | 38.38±0.04 | ^g^ | 52.32±0.16 | ^e^ | 20.19±0.08 | ^j^ | 28.15±0.08 | ^f^ | 2.44±0.01 | ^f^ | 6.22±0.01 | ^f^ | 17.95±0.04 | ^f^ | 40.09±0.08 | ^i^ | 2.64±0.03 | ^i^ |
| DK1 |  | 36.43±0.02 | ^h^ | 50.90±0.12 | ^ef^ | 20.72±0.06 | ^i^ | 28.46±0.06 | ^f^ | 2.58±0.03 | ^e^ | 6.58±0.05 | ^ef^ | 17.14±0.07 | ^gh^ | 40.93±0.40 | ^i^ | 3.99±0.06 | ^e^ |
| DK2 |  | 47.41±0.51 | ^d^ | 49.19±0.25 | ^f^ | 24.23±0.20 | ^f^ | 22.18±0.11 | ^k^ | 1.04±0.02 | ^l^ | 8.85±0.09 | ^c^ | 19.26±0.09 | ^d^ | 27.12±0.08 | ^k^ | 3.06±0.05 | ^g^ |
| DK9 |  | 35.48±0.30 | ^i^ | 37.28±0.26 | ^h^ | 17.18±0.13 | ^m^ | 17.44±0.08 | ^m^ | 0.89±0.02 | ^m^ | 6.51±0.03 | ^ef^ | 14.51±0.04 | ^jk^ | 20.69±0.17 | ^l^ | 2.18±0.01 | ^j^ |
| DK10 |  | 12.96±0.07 | ^n^ | 16.67±0.11 | ^j^ | 9.42±0.02 | ^p^ | 11.14±0.15 | ^o^ | 0.53±0.02 | ^o^ | 2.12±0.02 | ^k^ | 5.67±0.02 | ^m^ | 18.91±0.04 | ^m^ | 1.30±0.01 | ^l^ |
| PT |  | 52.02±0.15 | ^c^ | 70.23±0.58 | ^a^ | 31.72±0.19 | ^d^ | 37.43±0.16 | ^d^ | 1.34±0.01 | ^j^ | 8.88±0.11 | ^c^ | 23.89±0.15 | ^a^ | 57.52±0.35 | ^e^ | 3.89±0.06 | ^e^ |
| PT1 |  | 27.89±0.15 | ^l^ | 32.82±0.26 | ^i^ | 22.74±0.19 | ^g^ | 21.50±0.35 | ^l^ | 1.76±0.01 | ^h^ | 14.97±0.14 | ^a^ | 14.78±0.04 | ^j^ | 37.63±0.24 | ^j^ | 2.84±0.01 | ^h^ |
| PT2 |  | 27.73±0.24 | ^l^ | 40.30±0.07 | ^g^ | 28.97±0.09 | ^e^ | 29.09±0.02 | ^e^ | 1.49±0.04 | ^i^ | 2.41±0.84 | ^jk^ | 18.46±0.05 | ^e^ | 62.57±0.46 | ^d^ | 3.92±0.03 | ^e^ |
| PT9 |  | 39.38±0.61 | ^f^ | 60.99±0.30 | ^cd^ | 35.06±0.16 | ^c^ | 27.49±0.16 | ^g^ | 3.17±0.02 | ^d^ | 11.16±0.03 | ^b^ | 17.36±0.16 | ^g^ | 45.77±0.50 | ^g^ | 4.84±0.05 | ^c^ |
| PT10 |  | 14.96±0.07 | ^m^ | 11.05±0.03 | ^k^ | 9.72±0.01 | ^p^ | 7.97±0.04 | ^p^ | 0.53±0.01 | ^o^ | 2.80±0.02 | ^j^ | 5.73±0.02 | ^m^ | 11.25±0.04 | ^n^ | 1.16±0.01 | ^m^ |
| SN |  | 65.88±0.11 | ^a^ | 59.02±0.12 | ^d^ | 14.40±0.14 | ^o^ | 22.67±0.10 | ^k^ | 6.68±0.03 | ^a^ | 5.25±0.02 | ^g^ | 23.97±0.17 | ^a^ | 55.54±0.22 | ^f^ | 5.42±0.02 | ^b^ |
| SN1 |  | 53.96±0.06 | ^b^ | 52.85±0.04 | ^e^ | 19.51±0.37 | ^k^ | 25.44±0.08 | ^i^ | 4.64±0.04 | ^b^ | 4.54±0.03 | ^hi^ | 21.14±0.09 | ^c^ | 43.94±0.08 | ^h^ | 4.36±0.06 | ^d^ |
| SN2 |  | 35.77±0.27 | ^hi^ | 42.85±4.75 | ^g^ | 24.48±0.18 | ^f^ | 47.18±0.14 | ^a^ | 1.20±0.03 | ^k^ | 7.97±0.08 | ^d^ | 16.89±0.13 | ^h^ | 68.45±0.45 | ^c^ | 3.28±0.04 | ^f^ |
| SN9 |  | 47.79±0.25 | ^d^ | 53.27±0.48 | ^e^ | 21.46±0.34 | ^h^ | 24.57±0.22 | ^j^ | 3.37±0.07 | ^c^ | 6.88±0.13 | ^e^ | 19.30±0.24 | ^d^ | 40.95±0.26 | ^i^ | 3.87±0.08 | ^e^ |
| SN10 |  | 30.34±0.23 | ^k^ | 40.76±0.08 | ^g^ | 19.84±0.05 | ^jk^ | 21.50±0.17 | ^l^ | 1.32±0.03 | ^j^ | 4.94±0.05 | ^gi^ | 14.42±0.02 | ^k^ | 37.01±0.05 | ^j^ | 2.68±0.03 | ^i^ |
| AUR |  | 33.05±0.06 | ^j^ | 35.30±0.11 | ^hi^ | 16.15±0.01 | ^n^ | 16.15±0.10 | ^n^ | 0.75±0.01 | ^n^ | 6.23±0.03 | ^f^ | 13.79±0.20 | ^l^ | 20.25±0.10 | ^l^ | 2.01±0.03 | ^k^ |
| BRI |  | 39.51±0.52 | ^f^ | 63.70±0.40 | ^bc^ | 41.50±0.25 | ^a^ | 40.16±0.60 | ^b^ | 1.80±0.03 | ^h^ | 4.89±0.03 | ^gi^ | 23.31±0.01 | ^b^ | 90.96±0.17 | ^a^ | 5.62±0.15 | ^a^ |
| DRA |  | 40.77±0.26 | ^e^ | 65.12±0.11 | ^b^ | 40.51±0.27 | ^b^ | 38.67±0.31 | ^c^ | 2.10±0.02 | ^g^ | 5.07±0.02 | ^gh^ | 23.28±0.11 | ^b^ | 90.06±0.86 | ^b^ | 5.31±0.03 | ^b^ |
| EAR |  | 37.77±0.05 | ^g^ | 53.30±0.28 | ^e^ | 18.62±0.33 | ^l^ | 26.75±0.28 | ^h^ | 1.23±0.02 | ^k^ | 4.51±0.12 | ^i^ | 15.54±0.22 | ^i^ | 40.43±0.39 | ^i^ | 2.71±0.06 | ^hi^ |
| Mean±SD |  | 37.76±12.41 | | 46.73±15.34 | | 22.97±8.89 | | 26.00±9.66 | | 2.05±1.52 | | 6.36±3.04 | | 17.18±5.12 | | 44.74±21.68 | | 3.42±1.30 | |
| Min-Max |  | 12.88-63.03 | | 11.02-70.78 | | 9.41-41.82 | | 7.91-47.38 | | 0.51-6.71 | | 1.55-15.11 | | 5.63-24.19 | | 11.20-91.14 | | 1.48-5.77 | |
| CV% |  | 32.9 |  | 32.8 |  | 38.7 |  | 37.1 |  | 74.2 |  | 47.8 |  | 29.8 |  | 48.4 |  | 37.9 |  |

*different letters in the same column indicate statistically significant differences (P≤0.05); values are means ± standard deviation. The values are presented in [mg · 100 g^-1^ fresh matter].

**Supplementary Table 1B.** List of all 37 identified polyphenolic compounds in highbush blueberry (*Vaccinium corymbosum* L.) fruits, indicating the genotypes with the highest and lowest concentrations and the observed range across all genotypes (mg/100 g fresh weight).

| Compound | Highest (genotype, mg/100 g FW) | Lowest (genotype, mg/100 g FW) | Range (mg/100 g FW) |
| --- | --- | --- | --- |
| Caffeoyl-glucose (I) | PT10 (6.17) | PT9 (1.87) | 1.87 – 6.17 |
| Caffeoyl-glucose (II) | PT10 (2.08) | BRI/DRA (0.28) | 0.28 – 2.08 |
| Caffeoyl-glucose (III) | AUR (2.35) | DK2 (0.32) | 0.32 – 2.35 |
| Neochlorogenic acid | SN (4.46) | PT10 (0.40) | 0.40 – 4.46 |
| Chlorogenic acid | DK2 (157.97) | PT2 (53.08) | 53.08 – 157.97 |
| Cryptochlorogenic acid | SN (4.05) | SN10 (0.10) | 0.10 – 4.05 |
| Myricetin-3-galactoside | PT (6.52) | PT9 (1.26) | 1.26 – 6.52 |
| Quercetin diglucoside | PT10 (1.12) | PT9 (0.08) | 0.08 – 1.12 |
| Quercetin-3-rhamno-hexoside | BRI (4.40) | DK1 (0.21) | 0.21 – 4.40 |
| Quercetin-3-rutinoside | SN (2.80) | DK10 (0.36) | 0.36 – 2.80 |
| Quercetin-3-galactoside | PT (10.57) | PT10 (2.41) | 2.41 – 10.57 |
| Quercetin-3-methoxyhexoside | DK2 (3.21) | DK10 (1.43) | 1.43 – 3.21 |
| Quercetin-3-glucoside | DK9 (3.34) | PT1 (0.37) | 0.37 – 3.34 |
| Quercetin-3-arabinoside | DK2 (3.73) | DK10 (0.13) | 0.13 – 3.73 |
| Quercetin-3-caffeoylgalactoside | PT10 (1.03) | DK10 (0.15) | 0.15 – 1.03 |
| Quercetin-3-caffeoylglucoside | DK10 (5.58) | DK9 (0.10) | 0.10 – 5.58 |
| Quercetin-3-oxalylpentoside | DK2 (8.45) | PT1 (0.06) | 0.06 – 8.45 |
| Quercetin-3-rhamnoside | SN1 (1.24) | DK10 (0.25) | 0.25 – 1.24 |
| Quercetin-3-dimethoxyrhamnoside | DK2 (2.22) | DK (0.03) | 0.03 – 2.22 |
| Quercetin-3-(6’‑acetyl)galactoside (I) | SN (0.30) | DRA (0.02) | 0.02 – 0.30 |
| Quercetin-3-(6’‑acetyl)galactoside (II) | BRI (0.65) | DK/DK1/PT/PT1 (0.01) | 0.01 – 0.65 |
| Quercetin (aglycone) | SN (0.52) | DK9/AUR (0.01) | 0.01 – 0.52 |
| Procyanidin dimer (A) | DK1 (10.40) | PT10 (2.08) | 2.08 – 10.40 |
| Procyanidin dimer (B) | DK10 (7.52) | DK9 (1.43) | 1.43 – 7.52 |
| Catechin | DK2 (37.47) | DRA (4.26) | 4.26 – 37.47 |
| Procyanidin dimer (C) | PT10 (17.47) | DK10 (1.20) | 1.20 – 17.47 |
| (–)-Epicatechin | DK1 (8.23) | PT9 (1.42) | 1.42 – 8.23 |
| Procyanidin trimer | PT10 (2.85) | EAR (0.49) | 0.49 – 2.85 |
| Cyanidin‑3‑O‑glucoside | SN (65.88) | DK10 (12.96) | 12.96 – 65.88 |
| Delphinidin‑3‑O‑glucoside | PT (70.23) | PT10 (11.05) | 11.05 – 70.23 |
| Malvidin‑3‑O‑glucoside | BRI (41.50) | DK10 (9.42) | 9.42 – 41.50 |
| Petunidin‑3‑O‑glucoside | SN2 (47.18) | PT10 (7.97) | 7.97 – 47.18 |
| Delphinidin‑3‑arabinoside | SN (6.68) | DK10 (0.53) | 0.53 – 6.68 |
| Cyanidin‑3‑arabinoside | PT1 (14.97) | DK10 (2.12) | 2.12 – 14.97 |
| Petunidin‑3‑arabinoside | SN (23.97) | DK10 (5.67) | 5.67 – 23.97 |
| Malvidin‑3‑galactoside | BRI (90.96) | PT10 (11.25) | 11.25 – 90.96 |
| Malvidin‑3‑arabinoside | BRI (5.62) | PT10 (1.16) | 1.16 – 5.62 |

**Supplementary Table 2.** Correlation coefficients

|  | CIE L | Firmness | Puncture | Extract% | Acidicity | L-ascorbic acid | NO_3_^-^ | NO_2_^-^ | ABTS | DPPH | FRAP | Phenolic acid | Flavonols | Flavan-3-ols | Anthocyanins | Polyphenols |
| --- | --- | --- | --- | --- | --- | --- | --- | --- | --- | --- | --- | --- | --- | --- | --- | --- |
| CIE L | 1.00 | 0.01 | 0.20 | -0.27 | -0.10 | -0.17 | 0.23 | -0.12 | 0.10 | -0.22 | -0.09 | -0.20 | -**0.58** | -0.45 | -0.25 | -0.39 |
| Firmness | 0.01 | 1.00 | **0.59** | 0.42 | 0.20 | 0.31 | -0.07 | **-0.56** | 0.39 | 0.27 | **-0.62** | -0.39 | 0.08 | 0.10 | 0.32 | 0.21 |
| Puncture | 0.20 | **0.59** | 1.00 | 0.03 | 0.31 | 0.21 | 0.07 | -0.26 | 0.00 | -0.21 | **-0.63** | 0.09 | 0.06 | -0.34 | 0.44 | 0.40 |
| Extract% | -0.27 | 0.42 | 0.03 | 1.00 | 0.24 | 0.20 | 0.40 | -0.11 | **0.51** | 0.22 | -0.10 | -0.19 | 0.12 | 0.35 | -0.20 | -0.19 |
| Acidicity | -0.10 | 0.20 | 0.31 | 0.24 | 1.00 | -0.21 | **0.51** | 0.38 | 0.41 | 0.11 | -0.01 | 0.14 | -0.15 | -0.02 | -0.20 | -0.16 |
| L-ascorbic acid | -0.17 | 0.31 | 0.21 | 0.20 | -0.21 | 1.00 | -0.07 | **-0.50** | -0.21 | -0.21 | **-0.46** | 0.13 | 0.22 | -0.02 | **0.66** | **0.67** |
| NO_3_^-^ | 0.23 | -0.07 | 0.07 | 0.40 | **0.51** | -0.07 | 1.00 | 0.44 | 0.20 | -0.36 | -0.01 | 0.21 | -0.16 | -0.06 | -0.43 | -0.36 |
| NO_2_^-^ | -0.12 | **-0.56** | -0.26 | -0.11 | 0.38 | **-0.50** | 0.44 | 1.00 | -0.06 | -0.30 | **0.59** | **0.48** | -0.22 | -0.13 | **-0.60** | **-0.46** |
| ABTS | 0.10 | 0.39 | 0.00 | **0.51** | 0.41 | -0.21 | 0.20 | -0.06 | 1.00 | **0.67** | -0.02 | **-0.51** | -0.14 | -0.01 | -0.27 | -0.41 |
| DPPH | -0.22 | 0.27 | -0.21 | 0.22 | 0.11 | -0.21 | -0.36 | -0.30 | **0.67** | 1.00 | 0.19 | **-0.60** | -0.01 | 0.18 | -0.06 | -0.20 |
| FRAP | -0.09 | **-0.62** | **-0.63** | -0.10 | -0.01 | **-0.46** | -0.01 | **0.59** | -0.02 | 0.19 | 1.00 | 0.15 | -0.29 | 0.08 | **-0.68** | **-0.61** |
| Phenolic acid | -0.20 | -0.39 | 0.09 | -0.19 | 0.14 | 0.13 | 0.21 | **0.48** | **-0.51** | **-0.60** | 0.15 | 1.00 | 0.15 | 0.24 | -0.05 | 0.28 |
| Flavonols | **-0.58** | 0.08 | 0.06 | 0.12 | -0.15 | 0.22 | -0.16 | -0.22 | -0.14 | -0.01 | -0.29 | 0.15 | 1.00 | 0.45 | 0.45 | **0.58** |
| Flavan-3-ols | -0.45 | 0.10 | -0.34 | 0.35 | -0.02 | -0.02 | -0.06 | -0.13 | -0.01 | 0.18 | 0.08 | 0.24 | 0.45 | 1.00 | -0.19 | 0.05 |
| Anthocyanins | -0.25 | 0.32 | 0.44 | -0.20 | -0.20 | **0.66** | -0.43 | **-0.60** | -0.27 | -0.06 | **-0.68** | -0.05 | 0.45 | -0.19 | 1.00 | **0.93** |
| Polyphenols | -0.39 | 0.21 | 0.40 | -0.19 | -0.16 | **0.67** | -0.36 | **-0.46** | -0.41 | -0.20 | **-0.61** | 0.28 | **0.58** | 0.05 | **0.93** | 1.00 |

Correlation value presented in red is significant at the 0.05 level

**Supplementary Table 3.** Correlations coefficients

|  | CIE L | Firmness | Puncture | Extract (%) | Acidicity | ABTS | DPPH | FRAP | L-ascorbid acid | NO_3_^-^ | NO_2_^-^ | Caffeoyl-glucose1 | Caffeoyl-glucose2 |
| --- | --- | --- | --- | --- | --- | --- | --- | --- | --- | --- | --- | --- | --- |
| CIE L | **1.00** | 0.01 | 0.20 | -0.26 | -0.09 | 0.10 | -0.21 | -0.09 | -0.16 | 0.22 | -0.11 | -0.08 | 0.20 |
| Firmness | 0.01 | **1.00** | 0.54 | 0.42 | 0.19 | 0.39 | 0.27 | -0.61 | 0.31 | -0.07 | -0.55 | -0.08 | 0.10 |
| Puncture | 0.20 | 0.54 | **1.00** | 0.02 | 0.30 | 0.01 | -0.20 | -0.59 | 0.19 | 0.07 | -0.23 | -0.05 | 0.00 |
| Extract (%) | -0.26 | 0.42 | 0.02 | **1.00** | 0.23 | 0.50 | 0.22 | -0.10 | 0.20 | 0.39 | -0.11 | -0.41 | -0.20 |
| Acidicity | -0.09 | 0.19 | 0.30 | 0.23 | **1.00** | 0.41 | 0.11 | -0.02 | -0.20 | 0.50 | 0.38 | -0.15 | -0.12 |
| ABTS | 0.10 | 0.39 | 0.01 | 0.50 | 0.41 | **1.00** | 0.67 | -0.02 | -0.21 | 0.20 | -0.07 | -0.26 | -0.07 |
| DPPH | -0.21 | 0.27 | -0.20 | 0.22 | 0.11 | 0.67 | **1.00** | 0.19 | -0.21 | -0.36 | -0.29 | -0.26 | 0.01 |
| FRAP | -0.09 | -0.61 | -0.59 | -0.10 | -0.02 | -0.02 | 0.19 | **1.00** | -0.45 | -0.01 | 0.58 | 0.00 | -0.08 |
| L-ascorbid acid | -0.16 | 0.31 | 0.19 | 0.20 | -0.20 | -0.21 | -0.21 | -0.45 | **1.00** | -0.07 | -0.48 | -0.06 | -0.56 |
| NO_3_^-^ | 0.22 | -0.07 | 0.07 | 0.39 | 0.50 | 0.20 | -0.36 | -0.01 | -0.07 | **1.00** | 0.44 | -0.08 | -0.06 |
| NO_2_^-^ | -0.11 | -0.55 | -0.23 | -0.11 | 0.38 | -0.07 | -0.29 | 0.58 | -0.48 | 0.44 | **1.00** | 0.08 | -0.05 |
| Caffeoyl-glucose1 | -0.08 | -0.08 | -0.05 | -0.41 | -0.15 | -0.26 | -0.26 | 0.00 | -0.06 | -0.08 | 0.08 | **1.00** | 0.38 |
| Caffeoyl-glucose2 | 0.20 | 0.10 | 0.00 | -0.20 | -0.12 | -0.07 | 0.01 | -0.08 | -0.56 | -0.06 | -0.05 | 0.38 | **1.00** |
| Caffeoyl-glucose3 | -0.04 | 0.24 | -0.10 | 0.45 | 0.30 | 0.27 | 0.17 | 0.02 | 0.11 | 0.14 | -0.11 | -0.11 | 0.00 |
| Neochlorogenic acid | -0.06 | 0.31 | 0.06 | 0.16 | -0.09 | 0.06 | 0.32 | -0.13 | 0.28 | -0.17 | -0.44 | -0.11 | 0.19 |
| Chlorogenic acid | -0.20 | -0.22 | 0.14 | 0.01 | 0.11 | -0.33 | -0.47 | 0.11 | 0.15 | 0.19 | 0.39 | 0.10 | -0.04 |
| Cryptochlorogenic acid | 0.17 | 0.27 | -0.05 | -0.04 | -0.09 | 0.38 | 0.37 | -0.27 | -0.10 | -0.05 | -0.39 | 0.31 | 0.39 |
| Myricetin 3-galactoside | -0.24 | -0.26 | -0.09 | -0.46 | 0.09 | -0.12 | 0.12 | 0.37 | -0.44 | -0.25 | 0.45 | 0.47 | 0.31 |
| Quercetin diglucoside | 0.34 | 0.06 | 0.02 | -0.31 | 0.01 | -0.14 | -0.19 | -0.13 | -0.15 | 0.25 | -0.08 | 0.48 | 0.52 |
| Quercetin 3-rhamno-hexoside | 0.05 | 0.29 | 0.25 | 0.05 | -0.16 | -0.08 | -0.11 | -0.55 | 0.69 | -0.08 | -0.60 | -0.16 | -0.42 |
| Quercetin 3-rutinoside | 0.08 | 0.26 | 0.10 | 0.01 | -0.47 | -0.33 | -0.21 | -0.42 | 0.32 | -0.15 | -0.59 | 0.28 | 0.38 |
| Quercetin 3-galactoside | -0.45 | -0.30 | -0.05 | -0.32 | 0.11 | -0.13 | 0.07 | 0.29 | -0.29 | -0.28 | 0.46 | 0.29 | 0.04 |
| Quercetin 3-methoxyhexoside | -0.38 | 0.13 | 0.00 | 0.18 | -0.35 | -0.14 | 0.20 | -0.30 | 0.02 | -0.44 | -0.42 | -0.22 | 0.32 |
| Quercetin 3-glucoside | -0.24 | 0.22 | -0.16 | 0.60 | 0.20 | 0.37 | 0.35 | 0.18 | -0.08 | -0.05 | -0.04 | -0.25 | 0.05 |
| Quercetin 3-arabinoside | -0.19 | -0.03 | 0.17 | -0.26 | -0.22 | -0.41 | -0.34 | -0.36 | 0.50 | -0.09 | -0.21 | 0.13 | -0.29 |
| Quercetin 3-caffeoylgalactoside | 0.27 | -0.12 | -0.07 | -0.27 | -0.36 | -0.47 | -0.45 | -0.05 | -0.06 | 0.14 | -0.12 | 0.44 | 0.44 |
| Quercetin 3-caffeoylglucoside | 0.28 | 0.10 | 0.06 | 0.53 | 0.45 | 0.60 | 0.14 | -0.02 | -0.38 | 0.58 | 0.08 | -0.25 | 0.05 |
| Quercetin 3-oxalylpentoside | -0.38 | 0.12 | -0.01 | 0.22 | -0.37 | -0.24 | -0.15 | -0.26 | 0.59 | -0.02 | -0.32 | 0.11 | -0.19 |
| Quercetin 3-rhamnoside | 0.02 | 0.34 | 0.08 | -0.14 | -0.04 | 0.09 | 0.31 | -0.39 | -0.22 | -0.18 | -0.37 | -0.10 | 0.44 |
| Quercetin 3-dimethoxyrhamnoside | -0.16 | 0.06 | -0.07 | 0.47 | -0.14 | -0.02 | -0.06 | -0.14 | 0.30 | -0.04 | -0.28 | -0.46 | -0.12 |
| Quercetin 3-(6’-acetyl)galactoside | -0.26 | 0.22 | -0.18 | 0.50 | -0.19 | 0.07 | 0.24 | -0.11 | -0.07 | -0.19 | -0.33 | -0.35 | 0.36 |
| Quercetin 3-(6’-acetyl)galactoside | -0.03 | 0.41 | 0.30 | 0.25 | -0.11 | 0.04 | -0.17 | -0.61 | 0.58 | 0.06 | -0.50 | 0.05 | -0.16 |
| Quercetin | 0.36 | 0.11 | -0.07 | 0.05 | -0.20 | 0.17 | -0.01 | -0.13 | -0.44 | 0.13 | -0.15 | 0.30 | 0.64 |
| Procyanidin dimer1 | -0.42 | 0.22 | -0.27 | 0.36 | 0.14 | 0.34 | 0.60 | 0.20 | 0.21 | -0.25 | -0.23 | 0.05 | -0.17 |
| Procyanidin dimer2 | 0.06 | 0.16 | -0.06 | 0.07 | -0.25 | 0.32 | 0.30 | -0.26 | -0.01 | 0.00 | -0.36 | 0.25 | 0.07 |
| Catechin | -0.43 | 0.00 | -0.24 | 0.36 | 0.04 | -0.03 | 0.16 | 0.13 | -0.07 | -0.06 | 0.01 | -0.34 | 0.15 |
| Procyanidin dimer3 | 0.40 | -0.03 | 0.06 | -0.23 | -0.11 | -0.29 | -0.48 | -0.01 | -0.15 | 0.29 | 0.09 | 0.47 | 0.51 |
| (-)Epicatechin | -0.50 | 0.12 | -0.19 | 0.09 | 0.07 | 0.02 | 0.15 | -0.18 | 0.38 | -0.20 | -0.19 | 0.38 | -0.10 |
| Procyanidin trimer | -0.14 | 0.02 | -0.08 | -0.21 | -0.26 | -0.25 | -0.07 | -0.09 | -0.36 | -0.17 | -0.04 | 0.60 | 0.82 |
| Cyanidin-3-O-glucoside | -0.29 | 0.36 | 0.19 | 0.07 | -0.34 | -0.18 | 0.19 | -0.46 | 0.37 | -0.50 | -0.67 | 0.02 | 0.23 |
| Delphinidin-3-O-glucoside | -0.23 | 0.27 | 0.42 | -0.14 | -0.31 | -0.29 | -0.03 | -0.53 | 0.60 | -0.51 | -0.59 | 0.11 | -0.17 |
| Malvidin-3-O-glucoside | -0.15 | 0.18 | 0.50 | -0.29 | -0.11 | -0.27 | -0.24 | -0.64 | 0.56 | -0.34 | -0.37 | 0.12 | -0.39 |
| Petunidin-3-O-glucoside | -0.29 | 0.13 | 0.34 | -0.21 | 0.03 | -0.35 | -0.27 | -0.57 | 0.68 | -0.09 | -0.24 | 0.17 | -0.37 |
| Delphinidin 3-arabinoside | -0.01 | 0.40 | 0.05 | 0.07 | -0.38 | 0.07 | 0.41 | -0.36 | 0.11 | -0.44 | -0.71 | 0.04 | 0.41 |
| Cyanidin 3-arabinoside | -0.22 | -0.13 | 0.19 | -0.18 | 0.07 | -0.21 | -0.03 | -0.16 | -0.07 | -0.24 | 0.11 | -0.33 | 0.05 |
| Petunidin 3-arabinoside | -0.33 | 0.34 | 0.30 | -0.18 | -0.26 | -0.25 | 0.11 | -0.58 | 0.54 | -0.59 | -0.67 | 0.15 | -0.05 |
| Malvidin 3-galactoside | -0.11 | 0.31 | 0.38 | -0.26 | -0.06 | -0.15 | -0.10 | -0.67 | 0.64 | -0.24 | -0.49 | 0.25 | -0.29 |
| Malvidin 3-arabinoside | -0.10 | 0.42 | 0.38 | -0.13 | -0.32 | -0.13 | 0.11 | -0.73 | 0.52 | -0.48 | -0.78 | 0.13 | -0.03 |

Correlation value presented in red is significant at the 0.05 level

|  | Caffeoyl-glucose3 | Neochlorogenic acid | Chlorogenic acid | Cryptochlorogenic acid | Myricetin 3-galactoside | Quercetin diglucoside | Quercetin 3-rhamno-hexoside | Quercetin 3-rutinoside | Quercetin 3-galactoside | Quercetin 3-methoxyhexoside | Quercetin 3-glucoside | Quercetin 3-arabinoside | Quercetin 3-caffeoylgalactoside |
| --- | --- | --- | --- | --- | --- | --- | --- | --- | --- | --- | --- | --- | --- |
| CIE L | -0.04 | -0.06 | -0.20 | 0.17 | -0.24 | 0.34 | 0.05 | 0.08 | -0.45 | -0.38 | -0.24 | -0.19 | 0.27 |
| Firmness | 0.24 | 0.31 | -0.22 | 0.27 | -0.26 | 0.06 | 0.29 | 0.26 | -0.30 | 0.13 | 0.22 | -0.03 | -0.12 |
| Puncture | -0.10 | 0.06 | 0.14 | -0.05 | -0.09 | 0.02 | 0.25 | 0.10 | -0.05 | 0.00 | -0.16 | 0.17 | -0.07 |
| Extract% | 0.45 | 0.16 | 0.01 | -0.04 | -0.46 | -0.31 | 0.05 | 0.01 | -0.32 | 0.18 | 0.60 | -0.26 | -0.27 |
| Acidicity | 0.30 | -0.09 | 0.11 | -0.09 | 0.09 | 0.01 | -0.16 | -0.47 | 0.11 | -0.35 | 0.20 | -0.22 | -0.36 |
| ABTS | 0.27 | 0.06 | -0.33 | 0.38 | -0.12 | -0.14 | -0.08 | -0.33 | -0.13 | -0.14 | 0.37 | -0.41 | -0.47 |
| DPPH | 0.17 | 0.32 | -0.47 | 0.37 | 0.12 | -0.19 | -0.11 | -0.21 | 0.07 | 0.20 | 0.35 | -0.34 | -0.45 |
| FRAP | 0.02 | -0.13 | 0.11 | -0.27 | 0.37 | -0.13 | -0.55 | -0.42 | 0.29 | -0.30 | 0.18 | -0.36 | -0.05 |
| L-ascorbid acid | 0.11 | 0.28 | 0.15 | -0.10 | -0.44 | -0.15 | 0.69 | 0.32 | -0.29 | 0.02 | -0.08 | 0.50 | -0.06 |
| NO_3_^-^ | 0.14 | -0.17 | 0.19 | -0.05 | -0.25 | 0.25 | -0.08 | -0.15 | -0.28 | -0.44 | -0.05 | -0.09 | 0.14 |
| NO_2_^-^ | -0.11 | -0.44 | 0.39 | -0.39 | 0.45 | -0.08 | -0.60 | -0.59 | 0.46 | -0.42 | -0.04 | -0.21 | -0.12 |
| Caffeoyl-glucose1 | -0.11 | -0.11 | 0.10 | 0.31 | 0.47 | 0.48 | -0.16 | 0.28 | 0.29 | -0.22 | -0.25 | 0.13 | 0.44 |
| Caffeoyl-glucose2 | 0.00 | 0.19 | -0.04 | 0.39 | 0.31 | 0.52 | -0.42 | 0.38 | 0.04 | 0.32 | 0.05 | -0.29 | 0.44 |
| Caffeoyl-glucose3 | **1.00** | 0.25 | -0.07 | 0.13 | -0.28 | 0.10 | 0.05 | 0.15 | -0.44 | -0.13 | 0.80 | -0.60 | -0.13 |
| Neochlorogenic acid | 0.25 | **1.00** | -0.06 | 0.32 | 0.03 | 0.22 | 0.25 | 0.54 | -0.11 | 0.33 | 0.21 | -0.07 | 0.02 |
| Chlorogenic acid | -0.07 | -0.06 | **1.00** | -0.46 | 0.12 | -0.12 | -0.17 | -0.12 | 0.22 | -0.02 | -0.04 | 0.20 | -0.01 |
| Cryptochlorogenic acid | 0.13 | 0.32 | -0.46 | **1.00** | 0.00 | 0.66 | 0.25 | 0.42 | -0.27 | -0.05 | -0.12 | -0.04 | 0.32 |
| Myricetin 3-galactoside | -0.28 | 0.03 | 0.12 | 0.00 | **1.00** | 0.01 | -0.68 | -0.33 | 0.88 | -0.06 | -0.20 | -0.23 | -0.23 |
| Quercetin diglucoside | 0.10 | 0.22 | -0.12 | 0.66 | 0.01 | **1.00** | 0.19 | 0.54 | -0.36 | -0.29 | -0.30 | 0.09 | 0.76 |
| Quercetin 3-rhamno-hexoside | 0.05 | 0.25 | -0.17 | 0.25 | -0.68 | 0.19 | **1.00** | 0.54 | -0.58 | 0.05 | -0.17 | 0.63 | 0.28 |
| Quercetin 3-rutinoside | 0.15 | 0.54 | -0.12 | 0.42 | -0.33 | 0.54 | 0.54 | **1.00** | -0.47 | 0.36 | 0.02 | 0.26 | 0.70 |
| Quercetin 3-galactoside | -0.44 | -0.11 | 0.22 | -0.27 | 0.88 | -0.36 | -0.58 | -0.47 | **1.00** | 0.12 | -0.20 | -0.01 | -0.43 |
| Quercetin 3-methoxyhexoside | -0.13 | 0.33 | -0.02 | -0.05 | -0.06 | -0.29 | 0.05 | 0.36 | 0.12 | **1.00** | 0.20 | 0.12 | -0.05 |
| Quercetin 3-glucoside | 0.80 | 0.21 | -0.04 | -0.12 | -0.20 | -0.30 | -0.17 | 0.02 | -0.20 | 0.20 | **1.00** | -0.65 | -0.31 |
| Quercetin 3-arabinoside | -0.60 | -0.07 | 0.20 | -0.04 | -0.23 | 0.09 | 0.63 | 0.26 | -0.01 | 0.12 | -0.65 | **1.00** | 0.35 |
| Quercetin 3-caffeoylgalactoside | -0.13 | 0.02 | -0.01 | 0.32 | -0.23 | 0.76 | 0.28 | 0.70 | -0.43 | -0.05 | -0.31 | 0.35 | **1.00** |
| Quercetin 3-caffeoylglucoside | 0.34 | -0.19 | -0.34 | 0.24 | -0.32 | 0.08 | -0.10 | -0.14 | -0.35 | -0.17 | 0.32 | -0.46 | -0.04 |
| Quercetin 3-oxalylpentoside | -0.40 | 0.21 | 0.27 | 0.01 | -0.23 | -0.05 | 0.43 | 0.37 | -0.02 | 0.34 | -0.33 | 0.74 | 0.24 |
| Quercetin 3-rhamnoside | -0.04 | 0.07 | -0.41 | 0.47 | -0.20 | 0.42 | 0.25 | 0.31 | -0.34 | 0.34 | -0.10 | 0.14 | 0.32 |
| Quercetin 3-dimethoxyrhamnoside | 0.09 | 0.06 | 0.31 | -0.34 | -0.61 | -0.40 | 0.26 | 0.16 | -0.33 | 0.55 | 0.34 | 0.23 | -0.03 |
| Quercetin 3-(6’-acetyl)galactoside | 0.26 | 0.37 | -0.04 | -0.02 | -0.32 | -0.16 | 0.02 | 0.40 | -0.23 | 0.80 | 0.58 | -0.15 | 0.07 |
| Quercetin 3-(6’-acetyl)galactoside | 0.18 | 0.10 | 0.10 | 0.36 | -0.66 | 0.29 | 0.80 | 0.55 | -0.60 | 0.07 | -0.03 | 0.49 | 0.34 |
| Quercetin | 0.16 | 0.01 | -0.42 | 0.69 | -0.05 | 0.62 | -0.07 | 0.45 | -0.33 | 0.03 | 0.05 | -0.33 | 0.54 |
| Procyanidin dimer1 | 0.61 | 0.30 | -0.16 | 0.10 | 0.08 | -0.26 | -0.11 | -0.09 | 0.05 | 0.01 | 0.65 | -0.43 | -0.45 |
| Procyanidin dimer2 | -0.14 | -0.07 | -0.63 | 0.67 | -0.03 | 0.20 | 0.14 | 0.17 | -0.12 | -0.05 | -0.23 | 0.02 | 0.12 |
| Catechin | -0.02 | 0.13 | 0.30 | -0.33 | -0.19 | -0.28 | -0.09 | 0.03 | 0.02 | 0.62 | 0.34 | 0.14 | -0.01 |
| Procyanidin dimer3 | 0.03 | -0.07 | 0.29 | 0.29 | -0.12 | 0.78 | 0.05 | 0.45 | -0.40 | -0.28 | -0.22 | 0.12 | 0.82 |
| (-)Epicatechin | 0.18 | 0.21 | 0.06 | 0.22 | 0.14 | 0.00 | 0.16 | 0.14 | 0.22 | 0.09 | 0.14 | 0.19 | -0.14 |
| Procyanidin trimer | -0.29 | 0.12 | 0.08 | 0.32 | 0.45 | 0.41 | -0.36 | 0.39 | 0.32 | 0.43 | -0.17 | 0.05 | 0.46 |
| Cyanidin-3-O-glucoside | 0.04 | 0.71 | -0.12 | 0.17 | 0.00 | -0.06 | 0.32 | 0.64 | 0.05 | 0.74 | 0.15 | 0.18 | 0.01 |
| Delphinidin-3-O-glucoside | -0.14 | 0.44 | -0.02 | -0.01 | 0.04 | -0.21 | 0.46 | 0.42 | 0.17 | 0.44 | -0.13 | 0.37 | -0.13 |
| Malvidin-3-O-glucoside | -0.29 | -0.17 | 0.06 | -0.05 | -0.12 | -0.18 | 0.57 | 0.10 | 0.09 | 0.12 | -0.41 | 0.61 | -0.12 |
| Petunidin-3-O-glucoside | -0.06 | 0.20 | 0.09 | 0.01 | 0.02 | 0.00 | 0.53 | 0.22 | 0.12 | 0.02 | -0.33 | 0.50 | -0.11 |
| Delphinidin 3-arabinoside | 0.25 | 0.61 | -0.48 | 0.52 | 0.01 | 0.18 | 0.13 | 0.56 | -0.17 | 0.53 | 0.20 | -0.25 | 0.05 |
| Cyanidin 3-arabinoside | -0.25 | 0.02 | 0.36 | -0.36 | 0.30 | -0.37 | -0.21 | -0.29 | 0.44 | 0.51 | -0.15 | 0.05 | -0.43 |
| Petunidin 3-arabinoside | -0.10 | 0.48 | -0.15 | 0.17 | 0.02 | -0.05 | 0.53 | 0.51 | 0.12 | 0.52 | -0.11 | 0.44 | -0.03 |
| Malvidin 3-galactoside | -0.04 | 0.19 | -0.16 | 0.35 | -0.09 | 0.20 | 0.69 | 0.34 | -0.07 | -0.04 | -0.37 | 0.51 | 0.02 |
| Malvidin 3-arabinoside | -0.02 | 0.34 | -0.29 | 0.37 | -0.13 | 0.06 | 0.57 | 0.51 | -0.11 | 0.40 | -0.16 | 0.31 | 0.00 |

Correlation value presented in red is significant at the 0.05 level

|  | Quercetin 3-caffeoylglucoside | Quercetin 3-oxalylpentoside | Quercetin 3-rhamnoside | Quercetin 3-dimethoxyrhamnoside | Quercetin 3-(6’-acetyl)galactoside | Quercetin 3-(6’-acetyl)galactoside | Quercetin | Procyanidin dimer1 | Procyanidin dimer2 | Catechin | Procyanidin dimer3 | (-)Epicatechin | Procyanidin trimer |
| --- | --- | --- | --- | --- | --- | --- | --- | --- | --- | --- | --- | --- | --- |
| CIE L | 0.28 | -0.38 | 0.02 | -0.16 | -0.26 | -0.03 | 0.36 | -0.42 | 0.06 | -0.43 | 0.40 | -0.50 | -0.14 |
| Firmness | 0.10 | 0.12 | 0.34 | 0.06 | 0.22 | 0.41 | 0.11 | 0.22 | 0.16 | 0.00 | -0.03 | 0.12 | 0.02 |
| Puncture | 0.06 | -0.01 | 0.08 | -0.07 | -0.18 | 0.30 | -0.07 | -0.27 | -0.06 | -0.24 | 0.06 | -0.19 | -0.08 |
| Extract% | 0.53 | 0.22 | -0.14 | 0.47 | 0.50 | 0.25 | 0.05 | 0.36 | 0.07 | 0.36 | -0.23 | 0.09 | -0.21 |
| Acidicity | 0.45 | -0.37 | -0.04 | -0.14 | -0.19 | -0.11 | -0.20 | 0.14 | -0.25 | 0.04 | -0.11 | 0.07 | -0.26 |
| ABTS | 0.60 | -0.24 | 0.09 | -0.02 | 0.07 | 0.04 | 0.17 | 0.34 | 0.32 | -0.03 | -0.29 | 0.02 | -0.25 |
| DPPH | 0.14 | -0.15 | 0.31 | -0.06 | 0.24 | -0.17 | -0.01 | 0.60 | 0.30 | 0.16 | -0.48 | 0.15 | -0.07 |
| FRAP | -0.02 | -0.26 | -0.39 | -0.14 | -0.11 | -0.61 | -0.13 | 0.20 | -0.26 | 0.13 | -0.01 | -0.18 | -0.09 |
| L-ascorbic acid | -0.38 | 0.59 | -0.22 | 0.30 | -0.07 | 0.58 | -0.44 | 0.21 | -0.01 | -0.07 | -0.15 | 0.38 | -0.36 |
| NO_3_^-^ | 0.58 | -0.02 | -0.18 | -0.04 | -0.19 | 0.06 | 0.13 | -0.25 | 0.00 | -0.06 | 0.29 | -0.20 | -0.17 |
| NO_2_^-^ | 0.08 | -0.32 | -0.37 | -0.28 | -0.33 | -0.50 | -0.15 | -0.23 | -0.36 | 0.01 | 0.09 | -0.19 | -0.04 |
| Caffeoyl-glucose1 | -0.25 | 0.11 | -0.10 | -0.46 | -0.35 | 0.05 | 0.30 | 0.05 | 0.25 | -0.34 | 0.47 | 0.38 | 0.60 |
| Caffeoyl-glucose2 | 0.05 | -0.19 | 0.44 | -0.12 | 0.36 | -0.16 | 0.64 | -0.17 | 0.07 | 0.15 | 0.51 | -0.10 | 0.82 |
| Caffeoyl-glucose3 | 0.34 | -0.40 | -0.04 | 0.09 | 0.26 | 0.18 | 0.16 | 0.61 | -0.14 | -0.02 | 0.03 | 0.18 | -0.29 |
| Neochlorogenic acid | -0.19 | 0.21 | 0.07 | 0.06 | 0.37 | 0.10 | 0.01 | 0.30 | -0.07 | 0.13 | -0.07 | 0.21 | 0.12 |
| Chlorogenic acid | -0.34 | 0.27 | -0.41 | 0.31 | -0.04 | 0.10 | -0.42 | -0.16 | -0.63 | 0.30 | 0.29 | 0.06 | 0.08 |
| Cryptochlorogenic acid | 0.24 | 0.01 | 0.47 | -0.34 | -0.02 | 0.36 | 0.69 | 0.10 | 0.67 | -0.33 | 0.29 | 0.22 | 0.32 |
| Myricetin 3-galactoside | -0.32 | -0.23 | -0.20 | -0.61 | -0.32 | -0.66 | -0.05 | 0.08 | -0.03 | -0.19 | -0.12 | 0.14 | 0.45 |
| Quercetin diglucoside | 0.08 | -0.05 | 0.42 | -0.40 | -0.16 | 0.29 | 0.62 | -0.26 | 0.20 | -0.28 | 0.78 | 0.00 | 0.41 |
| Quercetin 3-rhamno-hexoside | -0.10 | 0.43 | 0.25 | 0.26 | 0.02 | 0.80 | -0.07 | -0.11 | 0.14 | -0.09 | 0.05 | 0.16 | -0.36 |
| Quercetin 3-rutinoside | -0.14 | 0.37 | 0.31 | 0.16 | 0.40 | 0.55 | 0.45 | -0.09 | 0.17 | 0.03 | 0.45 | 0.14 | 0.39 |
| Quercetin 3-galactoside | -0.35 | -0.02 | -0.34 | -0.33 | -0.23 | -0.60 | -0.33 | 0.05 | -0.12 | 0.02 | -0.40 | 0.22 | 0.32 |
| Quercetin 3-methoxyhexoside | -0.17 | 0.34 | 0.34 | 0.55 | 0.80 | 0.07 | 0.03 | 0.01 | -0.05 | 0.62 | -0.28 | 0.09 | 0.43 |
| Quercetin 3-glucoside | 0.32 | -0.33 | -0.10 | 0.34 | 0.58 | -0.03 | 0.05 | 0.65 | -0.23 | 0.34 | -0.22 | 0.14 | -0.17 |
| Quercetin 3-arabinoside | -0.46 | 0.74 | 0.14 | 0.23 | -0.15 | 0.49 | -0.33 | -0.43 | 0.02 | 0.14 | 0.12 | 0.19 | 0.05 |
| Quercetin 3-caffeoylgalactoside | -0.04 | 0.24 | 0.32 | -0.03 | 0.07 | 0.34 | 0.54 | -0.45 | 0.12 | -0.01 | 0.82 | -0.14 | 0.46 |
| Quercetin 3-caffeoylglucoside | **1.00** | -0.37 | 0.02 | 0.03 | 0.12 | 0.01 | 0.51 | -0.01 | 0.31 | -0.08 | -0.01 | -0.26 | -0.18 |
| Quercetin 3-oxalylpentoside | -0.37 | **1.00** | -0.07 | 0.44 | 0.20 | 0.47 | -0.24 | -0.07 | 0.12 | 0.37 | 0.05 | 0.38 | 0.24 |
| Quercetin 3-rhamnoside | 0.02 | -0.07 | **1.00** | -0.03 | 0.31 | 0.27 | 0.38 | -0.20 | 0.23 | 0.16 | 0.19 | -0.17 | 0.27 |
| Quercetin 3-dimethoxyrhamnoside | 0.03 | 0.44 | -0.03 | **1.00** | 0.69 | 0.35 | -0.24 | 0.01 | -0.37 | 0.77 | -0.06 | 0.04 | -0.09 |
| Quercetin 3-(6’-acetyl)galactoside | 0.12 | 0.20 | 0.31 | 0.69 | **1.00** | 0.12 | 0.18 | 0.17 | -0.18 | 0.77 | -0.09 | 0.07 | 0.30 |
| Quercetin 3-(6’-acetyl)galactoside | 0.01 | 0.47 | 0.27 | 0.35 | 0.12 | **1.00** | 0.14 | -0.08 | 0.15 | 0.00 | 0.35 | 0.23 | -0.09 |
| Quercetin | 0.51 | -0.24 | 0.38 | -0.24 | 0.18 | 0.14 | **1.00** | -0.20 | 0.52 | -0.26 | 0.49 | -0.19 | 0.48 |
| Procyanidin dimer1 | -0.01 | -0.07 | -0.20 | 0.01 | 0.17 | -0.08 | -0.20 | **1.00** | 0.11 | 0.07 | -0.41 | 0.59 | -0.14 |
| Procyanidin dimer2 | 0.31 | 0.12 | 0.23 | -0.37 | -0.18 | 0.15 | 0.52 | 0.11 | **1.00** | -0.47 | -0.10 | 0.13 | 0.16 |
| Catechin | -0.08 | 0.37 | 0.16 | 0.77 | 0.77 | 0.00 | -0.26 | 0.07 | -0.47 | **1.00** | -0.07 | 0.11 | 0.23 |
| Procyanidin dimer3 | -0.01 | 0.05 | 0.19 | -0.06 | -0.09 | 0.35 | 0.49 | -0.41 | -0.10 | -0.07 | **1.00** | -0.16 | 0.42 |
| (-)Epicatechin | -0.26 | 0.38 | -0.17 | 0.04 | 0.07 | 0.23 | -0.19 | 0.59 | 0.13 | 0.11 | -0.16 | **1.00** | 0.22 |
| Procyanidin trimer | -0.18 | 0.24 | 0.27 | -0.09 | 0.30 | -0.09 | 0.48 | -0.14 | 0.16 | 0.23 | 0.42 | 0.22 | **1.00** |
| Cyanidin-3-O-glucoside | -0.34 | 0.39 | 0.19 | 0.25 | 0.52 | 0.21 | -0.02 | 0.22 | 0.08 | 0.23 | -0.25 | 0.30 | 0.32 |
| Delphinidin-3-O-glucoside | -0.47 | 0.38 | -0.06 | 0.05 | 0.03 | 0.28 | -0.27 | 0.12 | 0.11 | -0.16 | -0.35 | 0.29 | 0.02 |
| Malvidin-3-O-glucoside | -0.37 | 0.29 | 0.07 | 0.00 | -0.31 | 0.48 | -0.32 | -0.16 | 0.12 | -0.29 | -0.21 | 0.22 | -0.17 |
| Petunidin-3-O-glucoside | -0.43 | 0.31 | -0.04 | -0.18 | -0.33 | 0.35 | -0.38 | 0.03 | 0.08 | -0.33 | -0.23 | 0.41 | -0.17 |
| Delphinidin 3-arabinoside | -0.03 | 0.01 | 0.41 | -0.05 | 0.42 | 0.09 | 0.42 | 0.32 | 0.41 | -0.09 | -0.14 | 0.09 | 0.32 |
| Cyanidin 3-arabinoside | -0.29 | -0.03 | 0.11 | 0.21 | 0.17 | -0.22 | -0.36 | -0.25 | -0.45 | 0.30 | -0.32 | -0.15 | 0.08 |
| Petunidin 3-arabinoside | -0.48 | 0.39 | 0.22 | 0.07 | 0.16 | 0.34 | -0.19 | 0.17 | 0.15 | -0.02 | -0.31 | 0.42 | 0.15 |
| Malvidin 3-galactoside | -0.31 | 0.27 | 0.16 | -0.24 | -0.36 | 0.55 | -0.11 | 0.04 | 0.33 | -0.47 | -0.10 | 0.42 | -0.13 |
| Malvidin 3-arabinoside | -0.27 | 0.27 | 0.32 | -0.04 | 0.06 | 0.49 | 0.07 | 0.13 | 0.40 | -0.28 | -0.20 | 0.34 | 0.09 |

Correlation value presented in red is significant at the 0.05 level

|  | Cyanidin-3-O-glucoside | Delphinidin-3-O-glucoside | Malvidin-3-O-glucoside | Petunidin-3-O-glucoside | Delphinidin 3-arabinoside | Cyanidin 3-arabinoside | Petunidin 3-arabinoside | Malvidin 3-galactoside | Malvidin 3-arabinoside |
| --- | --- | --- | --- | --- | --- | --- | --- | --- | --- |
| CIE L | -0.29 | -0.23 | -0.15 | -0.29 | -0.01 | -0.22 | -0.33 | -0.11 | -0.10 |
| Firmness | 0.36 | 0.27 | 0.18 | 0.13 | 0.40 | -0.13 | 0.34 | 0.31 | 0.42 |
| Puncture | 0.19 | 0.42 | 0.50 | 0.34 | 0.05 | 0.19 | 0.30 | 0.38 | 0.38 |
| Extract% | 0.07 | -0.14 | -0.29 | -0.21 | 0.07 | -0.18 | -0.18 | -0.26 | -0.13 |
| Acidicity | -0.34 | -0.31 | -0.11 | 0.03 | -0.38 | 0.07 | -0.26 | -0.06 | -0.32 |
| ABTS | -0.18 | -0.29 | -0.27 | -0.35 | 0.07 | -0.21 | -0.25 | -0.15 | -0.13 |
| DPPH | 0.19 | -0.03 | -0.24 | -0.27 | 0.41 | -0.03 | 0.11 | -0.10 | 0.11 |
| FRAP | -0.46 | -0.53 | -0.64 | -0.57 | -0.36 | -0.16 | -0.58 | -0.67 | -0.73 |
| L-ascorbic acid | 0.37 | 0.60 | 0.56 | 0.68 | 0.11 | -0.07 | 0.54 | 0.64 | 0.52 |
| NO_3_^-^ | -0.50 | -0.51 | -0.34 | -0.09 | -0.44 | -0.24 | -0.59 | -0.24 | -0.48 |
| NO_2_^-^ | -0.67 | -0.59 | -0.37 | -0.24 | -0.71 | 0.11 | -0.67 | -0.49 | -0.78 |
| Caffeoyl-glucose1 | 0.02 | 0.11 | 0.12 | 0.17 | 0.04 | -0.33 | 0.15 | 0.25 | 0.13 |
| Caffeoyl-glucose2 | 0.23 | -0.17 | -0.39 | -0.37 | 0.41 | 0.05 | -0.05 | -0.29 | -0.03 |
| Caffeoyl-glucose3 | 0.04 | -0.14 | -0.29 | -0.06 | 0.25 | -0.25 | -0.10 | -0.04 | -0.02 |
| Neochlorogenic acid | 0.71 | 0.44 | -0.17 | 0.20 | 0.61 | 0.02 | 0.48 | 0.19 | 0.34 |
| Chlorogenic acid | -0.12 | -0.02 | 0.06 | 0.09 | -0.48 | 0.36 | -0.15 | -0.16 | -0.29 |
| Cryptochlorogenic acid | 0.17 | -0.01 | -0.05 | 0.01 | 0.52 | -0.36 | 0.17 | 0.35 | 0.37 |
| Myricetin 3-galactoside | 0.00 | 0.04 | -0.12 | 0.02 | 0.01 | 0.30 | 0.02 | -0.09 | -0.13 |
| Quercetin diglucoside | -0.06 | -0.21 | -0.18 | 0.00 | 0.18 | -0.37 | -0.05 | 0.20 | 0.06 |
| Quercetin 3-rhamno-hexoside | 0.32 | 0.46 | 0.57 | 0.53 | 0.13 | -0.21 | 0.53 | 0.69 | 0.57 |
| Quercetin 3-rutinoside | 0.64 | 0.42 | 0.10 | 0.22 | 0.56 | -0.29 | 0.51 | 0.34 | 0.51 |
| Quercetin 3-galactoside | 0.05 | 0.17 | 0.09 | 0.12 | -0.17 | 0.44 | 0.12 | -0.07 | -0.11 |
| Quercetin 3-methoxyhexoside | 0.74 | 0.44 | 0.12 | 0.02 | 0.53 | 0.51 | 0.52 | -0.04 | 0.40 |
| Quercetin 3-glucoside | 0.15 | -0.13 | -0.41 | -0.33 | 0.20 | -0.15 | -0.11 | -0.37 | -0.16 |
| Quercetin 3-arabinoside | 0.18 | 0.37 | 0.61 | 0.50 | -0.25 | 0.05 | 0.44 | 0.51 | 0.31 |
| Quercetin 3-caffeoylgalactoside | 0.01 | -0.13 | -0.12 | -0.11 | 0.05 | -0.43 | -0.03 | 0.02 | 0.00 |
| Quercetin 3-caffeoylglucoside | -0.34 | -0.47 | -0.37 | -0.43 | -0.03 | -0.29 | -0.48 | -0.31 | -0.27 |
| Quercetin 3-oxalylpentoside | 0.39 | 0.38 | 0.29 | 0.31 | 0.01 | -0.03 | 0.39 | 0.27 | 0.27 |
| Quercetin 3-rhamnoside | 0.19 | -0.06 | 0.07 | -0.04 | 0.41 | 0.11 | 0.22 | 0.16 | 0.32 |
| Quercetin 3-dimethoxyrhamnoside | 0.25 | 0.05 | 0.00 | -0.18 | -0.05 | 0.21 | 0.07 | -0.24 | -0.04 |
| Quercetin 3-(6’-acetyl)galactoside | 0.52 | 0.03 | -0.31 | -0.33 | 0.42 | 0.17 | 0.16 | -0.36 | 0.06 |
| Quercetin 3-(6’-acetyl)galactoside | 0.21 | 0.28 | 0.48 | 0.35 | 0.09 | -0.22 | 0.34 | 0.55 | 0.49 |
| Quercetin | -0.02 | -0.27 | -0.32 | -0.38 | 0.42 | -0.36 | -0.19 | -0.11 | 0.07 |
| Procyanidin dimer1 | 0.22 | 0.12 | -0.16 | 0.03 | 0.32 | -0.25 | 0.17 | 0.04 | 0.13 |
| Procyanidin dimer2 | 0.08 | 0.11 | 0.12 | 0.08 | 0.41 | -0.45 | 0.15 | 0.33 | 0.40 |
| Catechin | 0.23 | -0.16 | -0.29 | -0.33 | -0.09 | 0.30 | -0.02 | -0.47 | -0.28 |
| Procyanidin dimer3 | -0.25 | -0.35 | -0.21 | -0.23 | -0.14 | -0.32 | -0.31 | -0.10 | -0.20 |
| (-)Epicatechin | 0.30 | 0.29 | 0.22 | 0.41 | 0.09 | -0.15 | 0.42 | 0.42 | 0.34 |
| Procyanidin trimer | 0.32 | 0.02 | -0.17 | -0.17 | 0.32 | 0.08 | 0.15 | -0.13 | 0.09 |
| Cyanidin-3-O-glucoside | **1.00** | 0.80 | 0.28 | 0.41 | 0.75 | 0.22 | 0.86 | 0.39 | 0.69 |
| Delphinidin-3-O-glucoside | 0.80 | **1.00** | 0.71 | 0.72 | 0.49 | 0.24 | 0.92 | 0.70 | 0.83 |
| Malvidin-3-O-glucoside | 0.28 | 0.71 | **1.00** | 0.77 | 0.03 | 0.28 | 0.68 | 0.82 | 0.73 |
| Petunidin-3-O-glucoside | 0.41 | 0.72 | 0.77 | **1.00** | 0.13 | 0.20 | 0.71 | 0.88 | 0.66 |
| Delphinidin 3-arabinoside | 0.75 | 0.49 | 0.03 | 0.13 | **1.00** | 0.06 | 0.57 | 0.28 | 0.67 |
| Cyanidin 3-arabinoside | 0.22 | 0.24 | 0.28 | 0.20 | 0.06 | **1.00** | 0.22 | 0.01 | 0.14 |
| Petunidin 3-arabinoside | 0.86 | 0.92 | 0.68 | 0.71 | 0.57 | 0.22 | **1.00** | 0.75 | 0.87 |
| Malvidin 3-galactoside | 0.39 | 0.70 | 0.82 | 0.88 | 0.28 | 0.01 | 0.75 | **1.00** | 0.82 |
| Malvidin 3-arabinoside | 0.69 | 0.83 | 0.73 | 0.66 | 0.67 | 0.14 | 0.87 | 0.82 | **1.00** |

Correlation value presented in red is significant at the 0.05 level


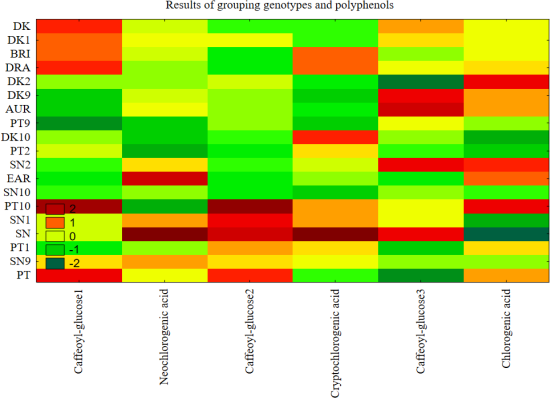


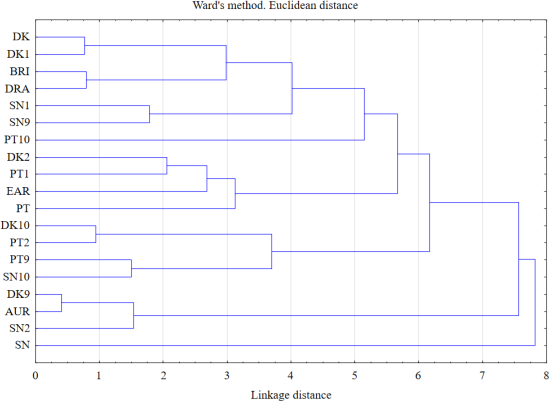

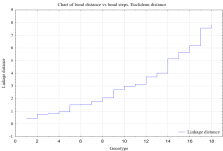

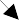


Phenolic acids


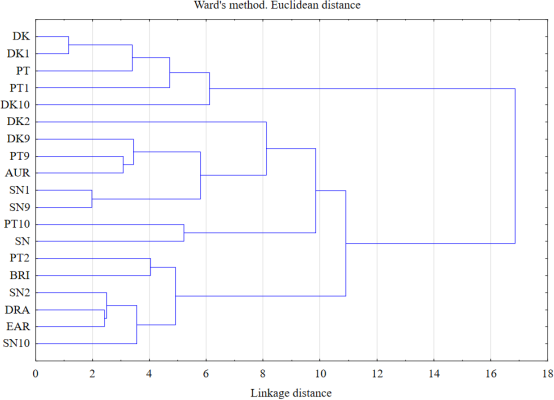

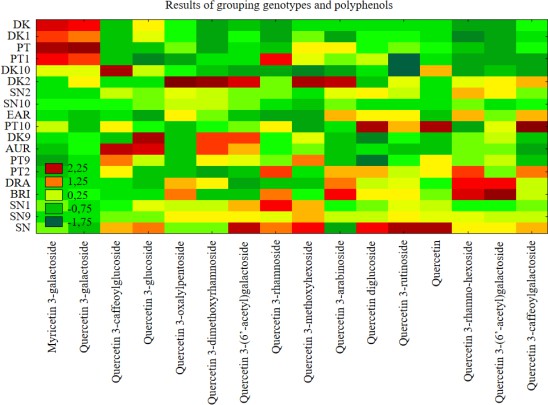

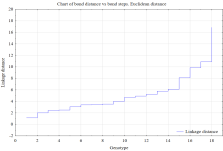

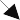


Flavonols


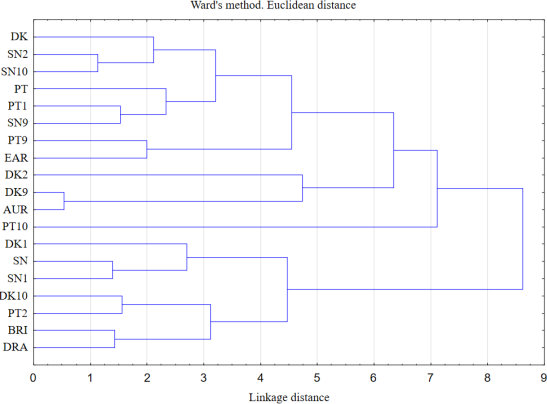

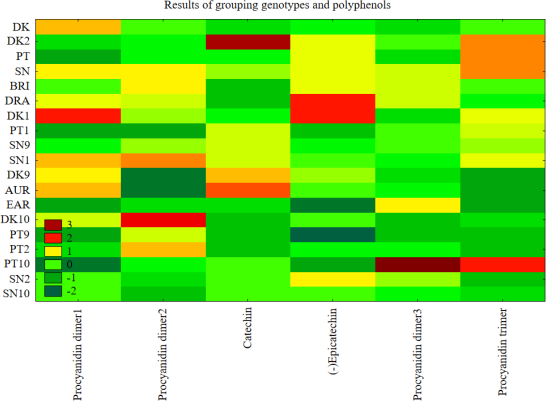

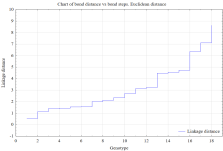

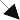

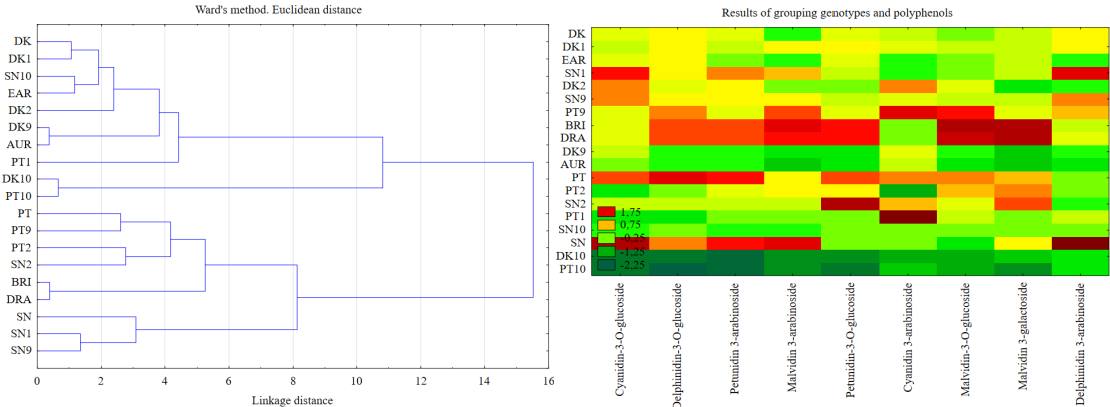

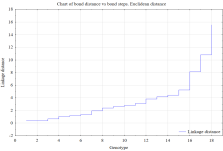

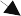


Flavan-3-ols

Anthocyanins

Supplementary Figure 1. Hierarchical clustering (Ward’s method, Euclidean distance) of 19 *Vaccinium corymbosum* L. genotypes based on (A) phenolic acids, (B) flavonols, (C) flavan-3-ols, and (D) anthocyanins. The vertical lines indicate the cuts-off used to form the Ward’s groups. On a heatmap displaying standardized data, the color of each cell corresponds to the standardized value (z-score), i.e., the number of standard deviations by which an observation departs from the variable’s mean. Positive values (shades of red and orange) indicate levels above the mean (e.g., z ≈ +1 is ~1 SD above the mean; z ≈ +2 denotes markedly elevated values), values near zero (yellows) indicate agreement with the mean, and negative values (green to dark green) indicate levels below the mean (e.g., z ≈ −1 is ~1 SD below the mean; z ≈ −2 denotes markedly reduced values). Dendrograms show linkage distance (Euclidean); vertical dashed lines mark the cluster cut-offs used to define groups G1–Gk. Genotype codes (e.g., BRI, DRA, DK, PT, SN, AUR, EAR) follow the abbreviations used in the manuscript.

distMDS-distPCA=0.1952, mantel.randtest r=0.8927, p-value=0.001


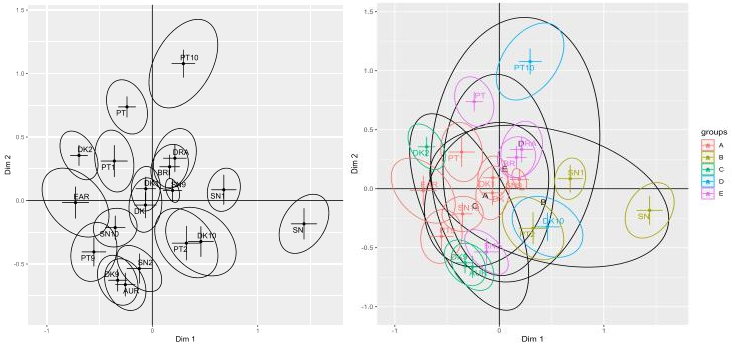

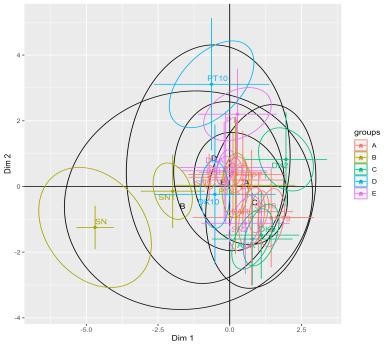


MDS without groups

MDS

PCA

17.035

distMDS-distEuclid=0.1747

distPCA-distEuclid=0.2489

22.413

22.413

stress=0.2238

stress=0.2145

stress=0.2145

stress=0.2238

Phenolic acids

distMDS-distPCA=0.1424, mantel.randtest r=0.9102, p-value=0.001


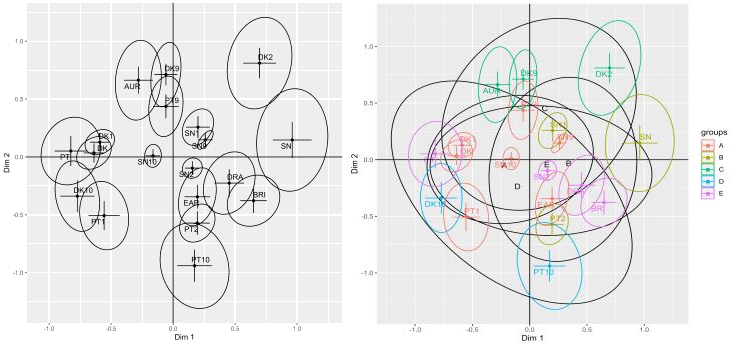

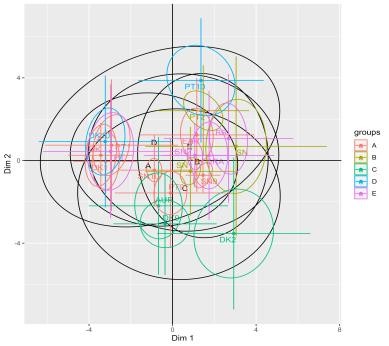


17.035

distMDS-distEuclid=0.1757

distPCA-distEuclid=0.2117

22.413

stress=0.2061

stress=0.2061

Flavan-3-ols

distMDS-distPCA=0.1463, mantel.randtest r=0.9192, p-value=0.001


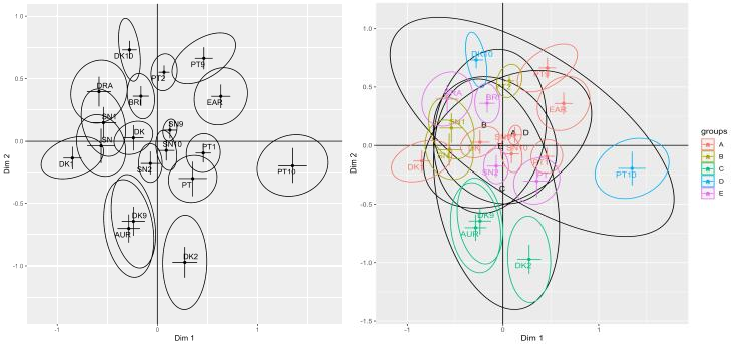

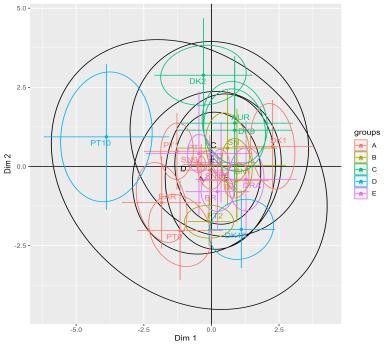


MDS without groups

MDS

PCA

17.035

distMDS-distEuclid=0.1619

distPCA-distEuclid=0.1932

Flavan-3-ols

22.413

stress=0.1234

stress=0.1234


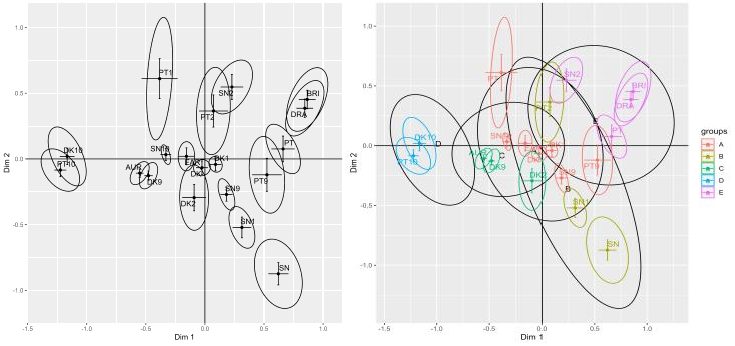

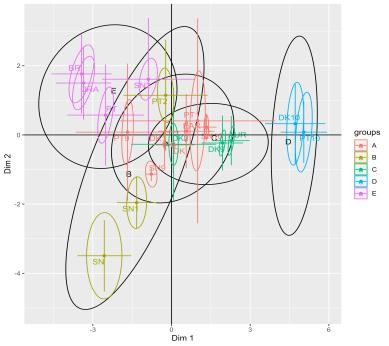


MDS without groups

MDS

PCA

17.035

distMDS-distEuclid=0.0918

distPCA-distEuclid=0.1337

Anthocyanins

distMDS-distPCA=0.0952, mantel.randtest r=0.9579, p-value=0.001

Supplementary Figure 2. MDS without groups - distribution of means in 2D space without group membership; MDS, PCA distribution of means in 2D space according to genotypes grouping as in Ward's method (as in Fig. 1).
